# Optimization of Field-Free Point Position, Gradient Field and Ferromagnetic Polymer Ratio for Enhanced Navigation of Magnetically Controlled Polymer-Based Microrobots in Blood Vessel

**DOI:** 10.3390/mi12040424

**Published:** 2021-04-13

**Authors:** Saqib Sharif, Kim Tien Nguyen, Doyeon Bang, Jong-Oh Park, Eunpyo Choi

**Affiliations:** 1 School of Mechanical Engineering, Chonnam National University, Gwangju 61186, Korea; saqibaan@gmail.com (S.S.); nguyenkimtien90@gmail.com (K.T.N.); 2 Korea Institute of Medical Microrobotics, Gwangju 61011, Korea; db@jnu.ac.kr; 3 College of AI Convergence, Chonnam National University, Gwangju 61186, Korea

**Keywords:** microrobots, field-free point, magnetic nanoparticle, 3D localization

## Abstract

Microscale and nanoscale robots, frequently referred to as future cargo systems for targeted drug delivery, can effectively convert magnetic energy into locomotion. However, navigating and imaging them within a complex colloidal vascular system at a clinical scale is exigent. Hence, a more precise and enhanced hybrid control navigation and imaging system is necessary. Magnetic particle imaging (MPI) has been successfully applied to visualize the ensemble of superparamagnetic nanoparticles (MNPs) with high temporal sensitivity. MPI uses the concept of field-free point (FFP) mechanism in the principal magnetic field. The gradient magnetic field (|∇B|) of MPI scanners can generate sufficient magnetic force in MNPs; hence, it has been recently used to navigate nanosized particles and micron-sized swimmers. In this article, we present a simulation analysis of the optimized navigation of an ensemble of microsized polymer MNP-based drug carriers in blood vessels. Initially, an ideal two-dimensional FFP case is employed for the basic optimization of the FFP position to achieve efficient navigation. Thereafter, a nine-coil electromagnetic actuation simulation system is developed to generate and manipulate the FFP position and |∇B|. Under certain vessel and fluid conditions, the particle trajectories of different ferromagnetic polymer ratios and |∇B| were compared to optimize the FFP position.

## 1. Introduction

Despite spectacular medical science innovations and studies in various advanced cancer treatments, cancer remains the second leading cause of death [1]. The conventional therapeutic methods include chemotherapy, radiation therapy, and photodynamic therapy (PDT). However, these traditional therapies have certain limitations; for example, in chemotherapy the entire body is exposed to high concentrations of toxic drugs, which cause severe damage to healthy tissue despite killing cancer cells; radiation therapy might induce severe side effects on the human body due to the radiation exposure. Moreover, PDT is expensive but it is also difficult to target cancer tissue for photosensitizing, and there is limited knowledge for clinical cancer treatment [2,3]. Chemotherapy is the most frequently used method for cancer treatment. There are several major classifications of anticancer drugs. Doxorubicin (DOX) is widely used to treat cancer as a broad-spectrum anticancer agent with an optimistically strong effect on solid tumors. DOX is a hydrophilic anthracycline antibiotic that, when inserted into the DNA of cancer cells, destroys the DNA double helix [4]. However, DOX not only acts on cancer cells but also badly affects normal cells and may also cause cardiac toxicity depending on the cumulative dose. Therefore, it is crucial to reduce toxicity to normal tissues without affecting its ability to kill cancer tissues. 

Targeted drug delivery (TDD) can precisely target and deliver an anticancer drug to infected tissue, which has emerged as a prominent solution to the toxicity problem [5,6]. A wide variety of both nano- and microscale carriers have been utilized as TDD vehicles. These carriers include both organic and inorganic materials. Some are created from metals, while others are made from polymers [3,7,8]. Different approaches have been suggested for steering these TDD vehicles; the use of acoustic radiation force, thermo-electromagnetically reacting bilayer-structured microrobots, octagram-shaped microgrippers, and macrophage-based microrobots have been proposed [9,10,11]. However, the use of electromagnetic actuators (EMAs) is the most widely utilized technique for navigating both nano- and microsized magnetic drug carriers [12,13,14]. The first human trial was reported in 1996 when cancer drugs were attached to iron core particles with diameters of 100 nm and then steered by an external magnet field to treat tumors [15]. 

Along with their biocompatibility, these small-scale carriers must satisfy several other requirements, such as the capability to move in different types of liquids and blood vessels. They must also be capable of overcoming obstacles and penetrating complex barriers. Finding carriers that can satisfy all these requisites is exigent, and the solutions formulated to resolve them usually require the combination of multiple materials on the same platform. For example, microrobots made of gelatin/polymer/hydrogel, encapsulating superparamagnetic nanoparticles (MNPs), and carrying DOX particles are potential candidates [16]. The magnetic properties of MNPs can be manipulated for navigation using external magnetic fields. First, the microrobot reaches a predetermined target location by the gradient magnetic field of the electromagnetic actuation system. Next, after near-infrared (NIR) irradiation, the gelatin/PVA of the microrobot is decomposed, and the MNPs and DOX drug particles are left in the target area. The disassembled MNPs are recovered from the target lesion by the magnetic field of the EMA system, only DOX particles remain in the target area to generate a therapeutic effect in the target lesion only. Therefore, MNP-mediated polymer-based carriers have emerged as one of the most promising cargo systems for TDD [3]. 

For efficient TDD, a real-time tracking of MNPs and microrobots in vivo is required. Several methods for developing a targeting scheme with feedback control, such as using ultrasound to locate solid microsized particles [17] or a microscope to track visible particles [18], have been investigated. However, none of the currently available clinical imaging modalities satisfies all the requirements. In the literature, the imaging aspect of magnetic nanoparticles has been thoroughly discussed, and the advantages and limitations of each imaging technique have been extensively described [19]. A major problem is to identify a specific navigation method that fits a particular imaging technique; a hybrid navigation and imaging method is highly preferable. The magnetic resonance navigation (MRN) system was developed based on magnetic resonance imaging (MRI) to steer and capture images of nanoparticles simultaneously using electromagnetic coils [20]; MRI is already clinically available and can provide relatively improved resolution and contrast of images. In vivo investigations of the MRN system were successfully conducted in the artery of a living animal using a magnetic bead with a diameter of 1.5 mm [21]. However, to date, the MRI of nanoscale and microscale particles has not been fully successful; thus, a new approach capable of both guiding and imaging magnetic nanoparticles should be conceived. 

Magnetic particle imaging (MPI) is a tracer-based (i.e., MNP-based) imaging technique that has gained considerable interest from MRI and TDD researchers [22]. Due to the unique magnetic features of MNPs, they can function at both the cellular and molecular levels. They can also produce a unique secondary magnetization signal as a reaction to an external magnetic field. Hence, they can suitably be used as contrast agents in MPI and drug carrier particles in TDD. Moreover, MPI scanners have been observed to achieve higher temporal and spatial resolutions and have high potentials for revolutionizing the field of biomedical imaging and TDD [23]. 

The fundamental principle of MPI is the detection of nonlinear responses of superparamagnetic iron oxide particles induced by a secondary oscillating magnetic field in a specific region, where the value of the primary magnetic field is zero—i.e., a field-free point (FFP). These FFPs can be generated and scanned by several combinations of permanent magnets and electromagnetic coils [24]. The simplest magnetic arrangement involves using four permanent magnets arranged in the form of a quadrupole to achieve static FFPs and an additional device to drive the FFP along a suitable trajectory [22,25]. The main disadvantage of this method is its low scanning speed. Other methods include the use of supportive drive coils that generate homogenous magnetic fields to drive a static FFP to a designated position [26]. The development of improved high-end MPI scanners has motivated TDD researchers to employ MPI modules for the navigation of magnetic carriers. Nothnagel et al. demonstrated this MPI capability by steering soft magnetic spheres by varying the FFP position using an MPI coil [27]. This somehow resolved one of the major problems encountered in an EMA system with MPI imaging—i.e., the problem of high-gradient fields interfering with the steering and tracking of nanoparticles in real time. However, the author did not consider any fluidic flow, MNPs are moved in stationary fluid. Recently, a hybrid guidance system capable of performing both the actuation and monitoring of nanoparticles was reported [28]. The paper proposed the stepwise navigation and two-dimensional (2D) tracking of nanoparticles in real time using a feedback control method. Simultaneous MPI imaging and MNP navigation in bifurcation flow experiments have also been recently demonstrated [29].

**Figure 1:**
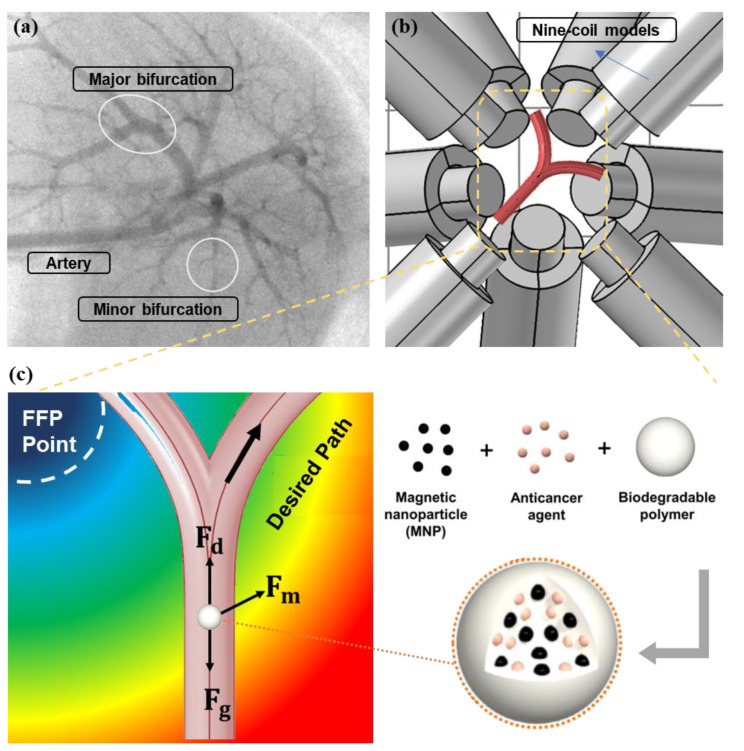
(**a**) Complexity of endovascular navigation in the portal vein of the rat. (**b**) Animated description of Y-shaped blood vessel bifurcation surrounded by a nine-coil electromagnetic actuators (EMA) system. (**c**) General concept of polymer-based microrobot with superparamagnetic nanoparticles (MNPs) along with free-body diagram of forces acting on microrobot steered by field-free point (FFP) position in blood vessel; $\vec{F_{d}},\;\vec{F_{m}},\;\mathrm{and}\;\vec{F_{g}}$ are the hydrodynamic drag, magnetic, and gravitational forces, respectively.

In this article, we present a simulation analysis of the optimized navigation of an ensemble of microsized polymer-MNP-based drug carriers in blood vessels (Figure 1). Initially, an ideal two-dimensional FFP case is employed for the basic optimization of the FFP position to achieve efficient navigation. Thereafter, a nine-coil electromagnetic actuation simulation system is developed to generate and manipulate the FFP position and |∇B|. Under certain vessel and fluid conditions, the trajectories of microrobots with different ferromagnetic polymer ratios and |∇B| were compared and optimized for efficient navigation.

## 2. Analytical Model

Consider a spherical ferromagnetic microrobot of radius *r* immersed in blood flowing in a vessel (Figure 1c). The microrobot is governed by several forces: magnetic force ($F_{m}$), blood hydrodynamic drag force ($F_{d}$), gravitational force ($F_{g}$), and contact force between MNPs and the vessel wall ($F_{c}$). The robot’s translational motion is given by

(1) $$m_{p}\frac{d\vec{v}}{dt}=\vec{F_{m}}+\vec{F_{d}}+\vec{F_{g}}+\vec{F_{c}},$$

where v is the translational velocity and $m_{p}$ is the robot mass. 

### 2.1. Hydrodynamic Drag Force

The low-velocity approximation of nonturbulent creeping flow was considered, and blood was assumed to be incompressible. Using the Stokes flow model, the hydrodynamic drag force ($F_{d}$) acting on a spherical body of radius *r* in the fluid is expressed as

(2) $$\vec{F_{d}}\;=\;\left(\frac{1}{\tau_{p}}\right)m_{p}\left(\vec{u}-\vec{v}\right)$$

where $m_{p}$ is the particle mass; $\tau_{p}\;$ is the particle velocity response time (SI unit: s); and u and v are the velocities of the fluid and robot, respectively. The particle velocity response time, however, depends on the flow conditions and parameters. The Reynolds number for laminar (Stokes) flow must satisfy Re≪1 to be valid; this number is given by

(3) $$Re\;=\;\frac{\rho_{p}\;\|\vec{u}-\vec{v}\|d_{p}}{\eta \;}$$

where η is the dynamic viscosity of the fluid, and $d_{p}$ is the diameter of microparticles. The particle velocity response time of spherical particles in a laminar flow is defined as follows:

(4) $$\tau_{p}\;=\;\frac{\rho_{p}\;d_{p}^{2}}{18\eta }$$

For a relatively moderate *Re* (i.e., 1<Re<800), the drag force can be set according to the Schiller–Naumann drag law [30]. Accordingly, the particle velocity response time is redefined as follows:

(5) $$\tau_{p}\;=\;\frac{4\rho_{p}\;d_{p}^{2}}{3\eta C_{D}Re\;}$$

where $C_{D}\;=\;\frac{24}{Re\;}\left(1+0.15\times Re^{0.687}\right)$.

### 2.2. Magnetic Force

The magnetic gradient induces a magnetic motive force on the nanoparticles and this process is known as magnetophoresis [31]. Assuming that the nanoparticles are homogeneous spheres with radius R and magnetization $\vec{M}$ in a magnetically linear fluid of permeability *μ*_1_. When subjected to a magnetic intensity $\vec{H}$, then the effective magnetic moment $m_{eff}$ is defined as:

(6) $${\vec{m}}_{eff}=4\pi R^{3}\left[\frac{\mu_{0}-\mu_{1}}{\mu_{0}+2\mu_{1}}\vec{H}+\frac{\mu_{0}}{\mu_{0}+2\mu_{1}}\vec{M}\right].$$

where $\mu_{0}=4\pi \times 10^{-7}N/A^{2}$ is the permeability of free space. The magnetophoretic force on a single MNP can be expressed in the gradient of the magnetic intensity ($\nabla \vec{H}$) and the effective magnetic dipole moment ($m_{eff}$) of the MNPs.

(7) $${\vec{F}}_{mnp}=2\pi \mu_{1}R^{3}\left[\frac{\mu_{0}-\mu_{1}}{\mu_{0}+2\mu_{1}}\nabla {\vec{H}}^{2}+\frac{2\mu_{0}}{\mu_{0}+2\mu_{1}}\vec{M}\left(H\right)\cdot \nabla \vec{H}\right]$$

The magnetization and magnetic intensity are linked by:

$$\vec{M}=\chi \vec{H}\;\;\;\;\&\;\;\;\vec{m}=4\pi R^{3}\chi \vec{H}.$$

where $\chi =\mu_{2}/\mu_{0}-1,$ is the magnetic susceptibility of the particles. In this case, Equation (7) simplifies to

(8) $${\vec{m}}_{eff}=4\pi R^{3}\frac{\mu_{2}-\mu_{1}}{\mu_{2}+2\mu_{1}}\vec{H}.$$

The magnetophoretic force can be expressed in the gradient of the magnetic intensity ($\nabla \vec{H}$) and the effective magnetic dipole moment ($m_{eff}$) of the MNPs

(9) $${\vec{F}}_{mnp}=\mu_{1}m_{eff}\nabla \vec{H}.$$

Combining Equations (8) and (9) for effective moment, the magnetic force on single MNP in a gradient magnetic field may be written as

(10) $${\vec{F}}_{mnp}=2\pi \mu_{1}R^{3}\frac{\mu_{2}-\mu_{1}}{\mu_{2}+2\mu_{1}}\vec{\nabla }H^{2}.$$

The nonlinear behavior in magnetic materials is very dominant and it cannot be ignored when modeling the electromechanics of magnetic particles. These materials can be classified as hard and soft magnetic materials. In both cases, the magnetization is dependent on magnetic field intensity. The most important phenomena in the nonlinear behavior of magnetic materials is the saturation, which limits the magnitude of the magnetization vector to a finite value, $M_{sat}$. Considering that the MNPs are magnetically soft, and the applied magnetic field is strong, the particles will saturate so that $\vec{M}\left(H\right)\;\to {\vec{M}}_{sat}$, and if $\mu_{1}≅\mu_{0}$, then the magnetic force simplifies to

(11) $${\vec{F}}_{mnp}=\;\frac{4}{3}\pi R^{3}\mu_{1}{\vec{M}}_{sat}.\nabla \vec{H}$$

Similarly, let us consider a spherical microrobot which is made of nonmagnetic binding polymer + MNPs. The magnetic force on this microparticle is defined by the quantity of MNPs in the microparticle. Therefore, if we define a volumetric ratio parameter, $\tau_{m}=\;\frac{V_{m}}{V}$, then the effective force on the microrobot will be modified as:

(12) $$\;{\vec{F}}_{m}=\;\tau_{m}V{\vec{M}}_{sat}.\nabla \vec{B}$$

where *V* and $\tau_{m}M_{sat}$ are the volume and saturation magnetization of the microrobot, respectively; $\vec{B}$ is the external magnetic field; and $\tau_{m}$ is the ferromagnetic ratio [32]. 

### 2.3. Gravitational Force

The gravitational force ($\vec{F_{g}}$) can be interpreted as the apparent weight—i.e., combined action of weight and fluid buoyancy:

(13) $$\vec{F_{g}}=V\left(\rho -\rho_{f}\right)\vec{g}$$

In the foregoing, $\rho \;=\;\tau_{m}\;\rho_{m}\;+\left(1\;–\;\tau_{m}\right)\rho_{poly}$, where $\rho_{m}$ and $\rho_{poly}$ are the densities of the magnetic material and polymer, respectively; $\rho_{f}$ is the carrier fluid density; and g is the gravitational acceleration.

### 2.4. Contact Force

The contact force results from particle–vessel collisions. This effect can be introduced in different ways, such as general reflection, elastic bouncing, and electrostatic attraction. Only the general reflection and bouncing effects have been considered in this current work. The robot velocity after collision is given by

(14) $$\vec{v}=\vec{v_{c}}-2\left(\vec{n}.{\vec{v}}_{c}\right)\vec{n}$$

where $v_{c}$ is the velocity of the robot before collision, and *n* is the normal vector on the wall surface. In order to reduce computational time, we have neglected of electrostatic interactions. 

## 3. Results and Discussion 

### 3.1. Ideal 2D Case

**Figure 2:**
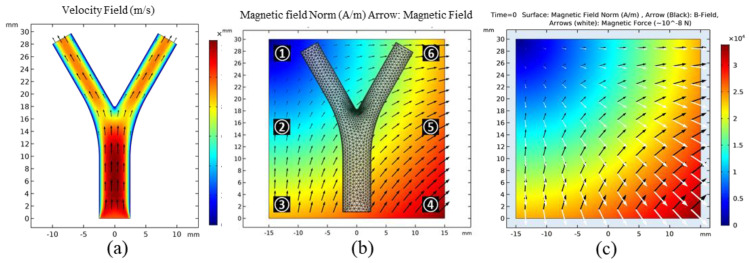
(**a**) Fluid velocity profiles in 2D vessel; (**b**) Meshed 2D bifurcation vessel along with FFP positions in the region of interest (ROI). (**c**) Comparison of magnetic and |∇B|.

The COMSOL Multiphysics^®^ software (5.4, COMSOL Inc, Burlington, MA, USA) was applied to perform all numerical modeling and animated simulation results with the table of animation list (Table S1) are in Supplementary Materials. The authors started with the simplest case, i.e., an ideal 2D FFP generation using a set of equations in the magnetic field, no current (mfnc) module. Then, the microrobots were navigated and tracked in 2D blood vessel laminar flow (spf) and particle tracing in fluid flow (fpt) modules consecutively. The vessel geometry was a 2D bifurcation with a diameter, total length, and bifurcation angle of 5 mm, 30 mm, and 40°, respectively (Figure 2b). A fluid initial velocity of 1 cm/s was set at the inlet, and the velocities at the outlets were controlled under suitable pressure conditions. The Reynolds number in a typical microfluidic channel is relatively small (Re < 1); hence, the flow can be considered to be laminar. The velocity profile of the developed flow is shown in Figure 2a.

The FFP strength and position were manually controlled using the mfnc module; for each FFP position, particle trajectories were traced and compared. Considering clinical limitations, we restricted our maximum gradient field to $\left|\nabla B\right|=1\;T/m\;\left(\sim 0.8\times 10^{6}\;A/m^{2}\right).$ An ensemble of 150 microrobots were released at the inlet with an initial velocity of 0.5 cm/s. These were released in three steps with 50 microrobots in each temporal step. Each microrobot has a diameter of 30 µm. Their magnetic properties can be controlled using the ferromagnetic ratio $(\tau_{m}$), which actually controls the volume concentration of MNPs in the binding polymer. In the 2D simulation, $\;\tau_{m}=0.2$ for most cases. A detailed comparison between the magnetic force and $\tau_{m}\;$ is explained in Section 3.2. 

**Figure 3:**
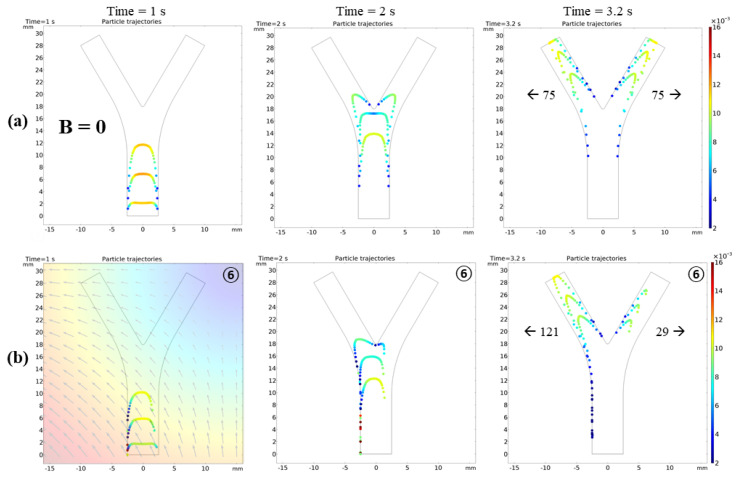
(**a**) Particle trajectories without any external magnetic field influence; (**b**) *FFP* at position **⑥**.

**Figure 4:**
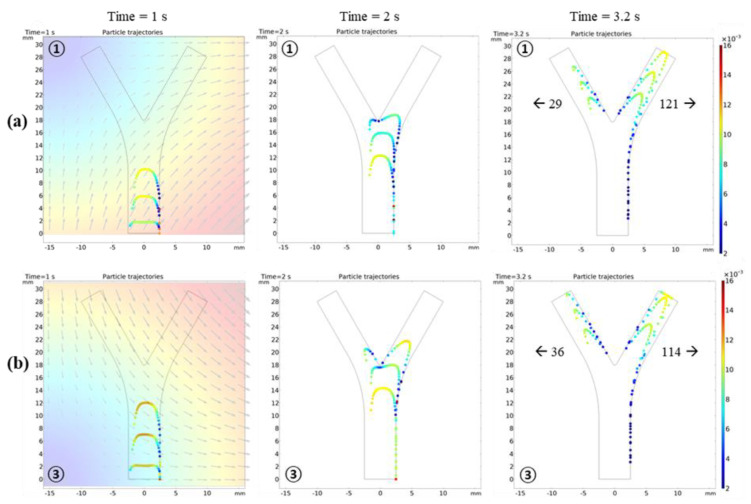
Particle trajectories under the influence of ∇B. (**a**) FFP positioned at top left corner **①**; ∇B has two components: $\nabla B_{x}$ forces microrobots toward right bifurcation and $\nabla B_{y}$ acts downward, decelerating and allowing microrobots to linger with $\nabla B_{x}$. (**b**) FFP positioned at lower left corner **③**; compared with to **①,** $\nabla B_{y}$ here is upward, accelerating the particles upward and leaving less time with $\nabla B_{x}$ (hence, it is less efficient).

Figure 3a shows that, without external forces, the robots simply follow the fluid flow with the drag force; hence, they are uniformly dispersed in the vessel. Using the particle counter feature of COMSOL, the exact number of microrobots reaching both outlets was monitored. In the absence of FFP, an equal number of microrobots, i.e., 75, reached each outlet with the same temporal pattern. When the FFP was applied, the microrobots reacted to the gradient field and their trajectories deviated. However, the trajectories are considerably dependent on the FFP position. Figure 2b shows the FFP positions used in the simulations. When the FFP was placed at position **⑥**, 80% of the microrobots were navigated to the left bifurcation (Figure 3b). We also simulated the trajectories of microrobots creeping towards right bifurcation when FFP is positioned at **①** and **③** (Figure 4). It is evident that $\nabla B_{x}$ forces microrobots toward right bifurcation while $\nabla B_{y}$ acts downward, decelerating and allowing microrobots to linger with $\nabla B_{x}$.

### 3.2. 3D Case

#### 3.2.1. FFP Generation

The COMSOL EMA simulation model the adoption of our experimentally validated EMA system; it has the same physical conditions as the coils (e.g., number of turns and scales) [33]. It can generate a magnetic field gradient of up to |∇B|~3 T/m, and each coil can support a maximum current of 20 A. The region of interest (ROI) in our system is $40\;\times \;40\;{\mathrm{mm}}^{2}$. This nine-coil EMA setup not only generates higher gradient fields but also provides the gradient field in the *z*-direction, thus minimizing the use of a mechanical microstage in most conventional four-coil EMA setups and can control the microrobots in 5-DOFs [34]. The detailed parameters of the EMA coil system are summarized in Table 1.

To measure the current (I) combination for a certain FFP at any given point P(x, y, z) in the workspace, the magnetic field created by the *n*th single electromagnetic coil can be expressed by vector $B_{n}\left(P\right)$. This is computed by the product of the input current (*i*) and magnetic field per unit current (${\hat{b}}_{n}\left(P\right)$), as follows:

(15) $$B_{n}\left(P\right)=\;\left[\;B_{x,n}\left(P\right)\;B_{y,n}\left(P\right)\;B_{z,n}\left(P\right)\;\right]=\left[\;{\hat{b}}_{x,n}\left(P\right)\;{\hat{b}}_{y,n}\left(P\right)\;{\hat{b}}_{z,n}\left(P\right)\;\right]i.$$

Using the superposition properties of electromagnetic coils, the resultant magnetic field at point *P* for the *n*-coil system can be expressed by a combination of linearly independent magnetic field vectors:

(16) $$B\left(P\right)=\left[{\hat{b}}_{x,1}\left(P\right)\;\cdots \;{\hat{b}}_{x,9}\left(P\right)\;{\hat{b}}_{y,1}\left(P\right)\;\cdots \;{\hat{b}}_{y,9}\left(P\right)\;{\hat{b}}_{z,1}\left(P\right)\;\cdots \;{\hat{b}}_{z,9}\left(P\right)\;\right]\left[\;i_{1}\;i_{2}\;\vdots \;i_{9}\;\right]\;=\hat{b}\left(P\right)i,$$

where $\hat{b}\left(P\right)\in R^{3\times 9}$ is the mapping matrix in tesla per ampere from the current input matrix (**i**) to the magnetic field. The partial derivative of the magnetic field (B) in the *x*, *y*, and *z* directions can similarly be derived to compute the magnetic force in Equation (14):

(17) $$\frac{\partial B\left(P\right)}{\partial q}=\left[\frac{\partial {\hat{b}}_{1}\left(P\right)}{\partial q}\;\ldots \;\frac{\partial {\hat{b}}_{9}\left(P\right)}{\partial q}\;\right]\left[\;i_{1}\;i_{2}\;\vdots \;i_{9}\;\right]=\frac{\partial \hat{b}\left(P\right)}{\partial q}i.$$

Equation (14) can be rewritten in the following matrix form:

(18) $$F_{m}=\tau_{m}V{\left[\frac{\partial B}{\partial x}\;\frac{\partial B}{\partial y}\;\frac{\partial B}{\partial z}\;\right]}^{T}M.$$

By combining Equations (16)–(18), the resultant magnetic field and magnetic force acing on the unit volume object can be calculated as

(19) $$D=\left[B\left(P\right)\;M^{T}G_{x}\left(P\right)\;M^{T}G_{y}\left(P\right)\;M^{T}z\left(P\right)\;\right]\left[\;i_{1}\;i_{2}\;\vdots \;i_{9}\;\right]=X_{u}\left(P\right)i,$$

where $D\;=\;{\left[B\;F\right]}^{T}$ is the desired matrix and $X_{u}\;\in R^{12\times 9}$ represents the conversion matrix corresponding to the nine coils in the ROI. To obtain the value of the input current in Equation (19), a matrix inversion was applied to $X_{u}$ to obtain the current matrix via the following equation, which is analogous to the methodology reported in [35]:

(20) $$i=X_{u}{}^{†}\left(P\right)D.$$

**Figure 5:**
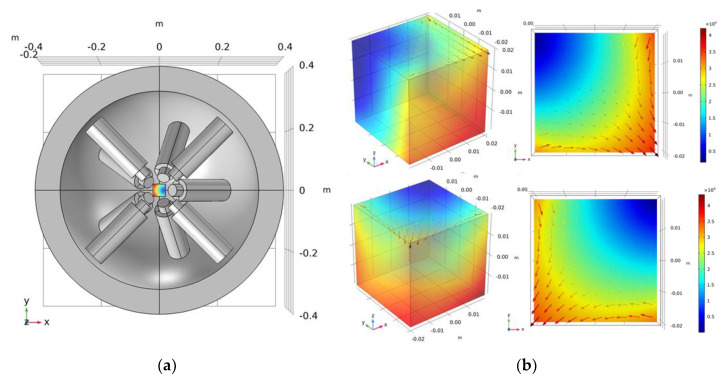
(**a**) Simulation model of proposed nine-coil EMA system; (**b**) simulated FFP obtained from COMSOL at predetermined positions.

With $B_{x}=B_{y}=B_{z}=0\;\mathrm{at}\;P\left(x,y,z\right)$, Maxwell’s equations, and *G_xx_* + *G_yy_* + *G_zz_* = 0, the suitable solution for *i* was obtained using the COMSOL built-in coefficient-based partial differential equation solver [25]. The simulation results of FFP at two different positions based on the proposed *i* solution are depicted in Figure 5b.

#### 3.2.2. Microrobot Navigation

**Figure 6:**
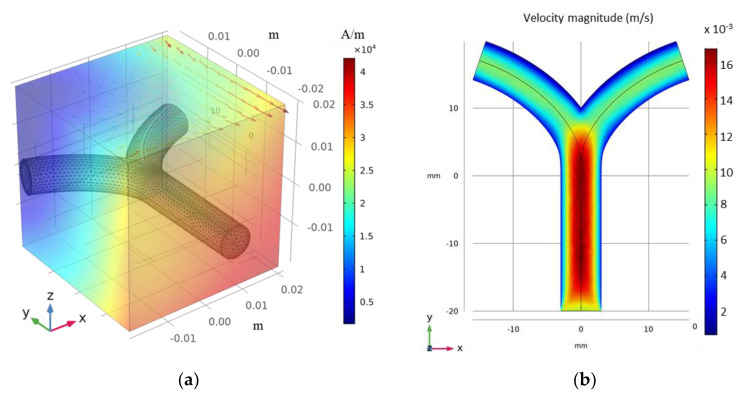
(**a**) Geometrical representation of meshed 3D bifurcation vessel in presence of FFP positioned at ① in the ROI; (**b**) XY projection of fluid velocity in 3D bifurcation vessel.

The vessel geometry is simply a modification of the 2D case with the same Y-shaped bifurcation (Figure 6a); the diameter, width, and height are 6, 30, and 40 mm, respectively. A maximum fluid velocity of 1.5 cm/s was set by controlling the pressure at the inlet and outlets. A nonpulsating steady creeping flow of incompressible fluid was considered. The cross-sectional velocity profile is shown in Figure 6b. In the case of blood, the viscosity (*η*) depends on both vessel diameter and hematocrit rate; however, in small vessels, its effect is virtually negligible [36].

**Figure 7:**
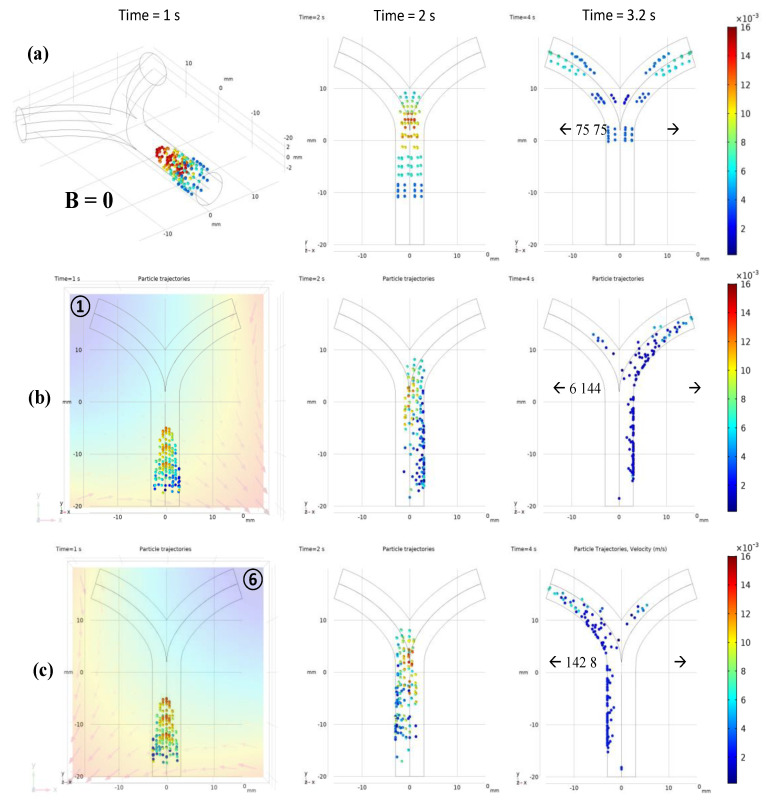
(**a**) Microrobot trajectories under the influence of ∇B in 3D blood vessel. (**a**) B = 0: microrobots are uniformly distributed on left and right bifurcation, each with 50% probability. (**b**) FFP positioned at top left corner, **①**: $\nabla B_{x}$ forces microrobots toward right bifurcation and $\nabla B_{y}$ decelerates microrobots, allowing them to linger with $\nabla B_{x}$. (**c**) FFP positioned at top right corner, **⑥**: $\nabla B_{x}$ forces microrobots toward left bifurcation and $\nabla B_{y}$ decelerates microrobots, allowing them to linger with $\nabla B_{x}$.

For a better comparison with 2D results, similar conditions were employed. All the animated simulation results of microrobot navigation in bifurcation flow with FFP field and the table of animation list (Table S1) are in Supplementary Materials. An ensemble of 150 microrobots were released at the inlet, each with an initial velocity of 0.5 cm/s. The microrobots were released in three steps with 50 microrobots in each temporal step. Each microrobot has a diameter of 30 μm, and the magnetic properties were controlled using the ferromagnetic ratio ($\tau_{m}$). Figure 7 shows the comparison of microrobot trajectories with and without FFP at certain positions for $\;\tau_{m}=0.5\;\mathrm{and}\;\nabla B=1\;T/m$. Different from the 2D simulations where the magnetic fields at points **①** and **⑥** are mirror images of each other, the ∇B generated by the EMA system is slightly different. The difference in ∇B was observed after checking the particle trajectories at both points in Figure 7. There were six and eight particles on the right bifurcation at points **①** and **⑥**, respectively, with a slight difference in velocities. This difference is caused by the five coils on the lower side that are not symmetrically arranged relative to these points, resulting in slightly different FFP patterns.

**Figure 8:**
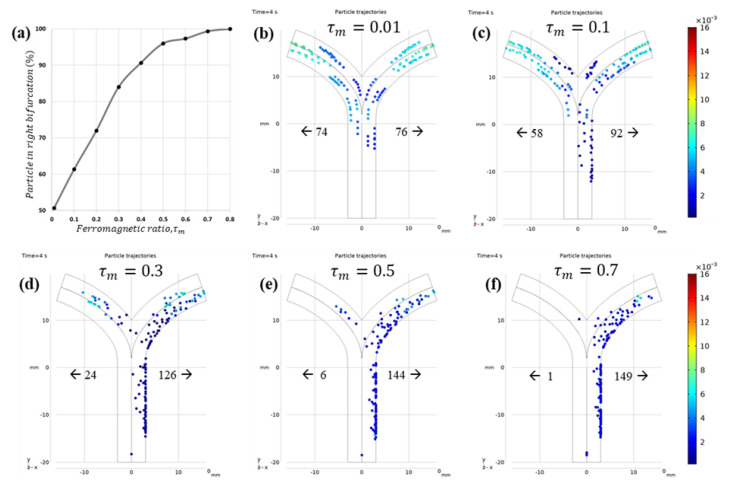
(**a**) Microrobots (%) flowing through required bifurcation and their dependence on $\;\tau_{m}$. (**b**–**f**) Microrobot trajectories under the influence of ∇B=1 T/m with FFP at point **①** and its dependence on $\;\tau_{m}$: (**b**) $\;\tau_{m}=0.01$, (**c**) $\;\tau_{m}=0.1$, (**d**) $\;\tau_{m}=0.3$, (**e**) $\;\tau_{m}=0.5$, and (**f**) $\;\tau_{m}=0.7$.

**Figure 9:**
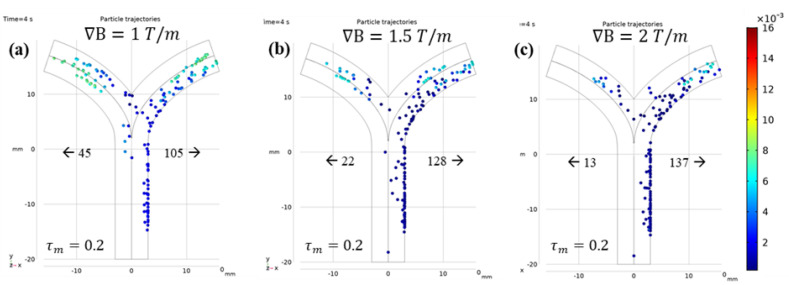
Microrobot trajectories with fixed $\tau_{m}=0.2\;$ under the influence of different ∇B  values: (**a**) ∇B=1 T/m, (**b**) ∇B=1.5 T/m, and (**c**) ∇B=2 T/m.

Figure 8 shows the dependence of navigation efficiency on $\tau_{m}$. With increasing $\;\tau_{m}$, the microrobots were observed to move slower as they dragged across the inner wall of the blood vessel. Although no significant particle–wall interactions were considered apart from general reflection, the accumulation of static particles on the wall cannot be ignored. Therefore, to achieve efficient navigation without blood vessel blockage, it is important to choose a combination of $\;\tau_{m}$ and ∇B suitable for the fluid flow conditions. In the literature, the reported average value of $\;\tau_{m}$ is between 0.1 and 0.2. The authors also attempted to find a suitable ∇B for $\;\tau_{m}=0.2$. The comparison of particle trajectories with ∇B=1.5 and 2 T/m is shown in Figure 9. 

## 4. Conclusions

Our simulation model was mainly designed for polymer-based microparticles, and we investigated the effect of FFP position, gradient field, and ferromagnetic ratio on navigation efficiency. We considered a relatively realistic approach to model blood–particle interactions in a 3D bifurcation vessel. The vessel diameter, blood velocity, and pressure were all realistic. The microcarriers were navigated in both directions using the FFP, and the navigation efficiency was improved for optimal gradient field and ferromagnetic ratio.

For realistic microparticle navigation in vivo experiments, all possible particle–particle and particle–wall interactions should be included in the simulation model. The role of frictional, Van der Waals, electrostatic, and steric contact forces will be vital and may result in the sticking of microparticle to vessel walls. The exact 3D blood map of each patient should be extracted using an imaging modality such as MPI/MRI. Subsequently, multiple FFPs at different bifurcations of blood vessels should be considered and investigated. Our future work will include all these interactions and multiple FFPs. We will then validate our model experimentally both in vitro and as well as in vivo. 

## Figures and Tables

**Table 1:** Simulation parameters.

Name	Expression	Value
Blood viscosity	$\eta_{plasma}$	$5\times 10^{-3}\;\left[\mathrm{Pa}\cdot s\right]$
Blood density	$\rho_{f}$	$1060\;\left[\mathrm{kg}\cdot m^{-3}\right]$
MNP density	$\rho_{m}$	$5200\;\left[\mathrm{kg}\cdot m^{-3}\right]$
Polymer density	$\rho_{poly}$	$1500\;\left[\mathrm{kg}\cdot m^{-3}\right]$
Robot diameter	$d_{p}$	30 [µm]
Saturation magnetization	$M_{s}$	$4\times 10^{5}\;\left[A\cdot m^{-1}\right]$
Vessel diameter (3D)	D	6 [mm]
Blood pressure (Max.)	P	50 [mm·Hg]
Initial velocity of blood	$v_{f_{0}}$	$1\;\left[\mathrm{cm}\cdot s^{-1}\right]$
Initial velocity ofrobot	$v_{0}$	$0.5\;\left[\mathrm{cm}\cdot s^{-1}\right]$
Turns/coil	n_coil	1400
Length of coil	l_coil	210 mm
Inner radius	r_min	21 mm
Outer radius	r_max	36 mm
Core	Pure Iron	Fe
Wires	Copper	Cu

## Data Availability

Not applicable.

## Supplementary Materials

The following are available online at https://www.mdpi.com/article/10.3390/mi12040424/s1, Animations: Animated simulation results of microrobot navigation in bifurcation flow with FFP field. Table S1: Descriptions of animation list (Table S1).
