# Supplementary material for: Optimization of Field-Free Point Position, Gradient Field and Ferromagnetic Polymer Ratio for Enhanced Navigation of Magnetically Controlled Polymer-Based Microrobots in Blood Vessel
Source: Micromachines (Basel). 2021 Apr 13;12(4):424. doi: 10.3390/mi12040424 (PMC8070347; doi:10.3390/mi12040424)
Supplement: Supplementary file 1 [file micromachines-12-00424-s001.zip › micromachines-1118489-SI.pdf]

micromachines

MDPI

Article

Supplementary Materials: Optimization of Field-Free Point Position, Gradient Field and Ferromagnetic Polymer Ratio for Enhanced Navigation of Magnetically Controlled Polymer-Based Microrobots in Blood Vessel

Saqib Sharif, Kim Tien Nguyen, Doyeon Bang, Jong-Oh Park and Eunpyo Choi

Table S1. Animated simulation results of microrobot navigation in bifurcation flow with FFP field.

Animation Reference	FFP Position	$\lvert\nabla B\rvert$ (T/m)	Ferromagnetic Ratio, τ_m	Description
Ani_2D_0	N/A	0	0.5	Particles flow through both branches equally, 75–75
Ani_2D_1	1	1	0.5	Particles are deviated toward *right* branch, 29–121
Ani_2D_2	2	1	0.5	Particles are deviated toward *right* branch, 31–129
Ani_2D_3	3	1	0.5	Particles are deviated toward *right* branch, 35–115
Ani_2D_4	4	1	0.5	Particles are deviated toward *left* branch, 115–35
Ani_2D_5	5	1	0.5	Particles are deviated toward *left* branch, 129–31
Ani_2D_6	6	1	0.5	Particles are deviated toward *left* branch, 121–29
Ani_3D_0	N/A	0	0.5	Particles flow through both branches equally, 75–75
Ani_3D_1	1	1	0.5	Particles are deviated toward *right* branch, 6–144
Ani_3D_2	6	1	0.5	Particles are deviated toward *left* branch, 142–8
Ani_3D_3	1	1	0.1	Particles are deviated toward *right* branch, 58–92
Ani_3D_4	1	1	0.2	Particles are deviated toward *right* branch, 42–108
Ani_3D_5	1	1	0.3	Particles are deviated toward *right* branch, 24–126
Ani_3D_6	1	1	0.4	Particles are deviated toward *right* branch, 14–136
Ani_3D_7	1	1	0.5	Particles are deviated toward *right* branch, 6–144
Ani_3D_8	1	1	0.6	Particles are deviated toward *right* branch, 4–146
Ani_3D_9	1	1	0.7	Particles are deviated toward *right* branch, 1–149
Ani_3D_10	1	1	0.8	Particles flow only through *right* branch, 0–150
Ani_3D_11	1	1.5	0.2	Particles are deviated toward *right* branch, 22–128
Ani_3D_12	1	2	0.2	Particles are deviated toward *right* branch, 13–137
