# Supplementary figures and images for: Optimization of Field-Free Point Position, Gradient Field and Ferromagnetic Polymer Ratio for Enhanced Navigation of Magnetically Controlled Polymer-Based Microrobots in Blood Vessel
Source: Micromachines (Basel). 2021 Apr 13;12(4):424. doi: 10.3390/mi12040424 (PMC8070347; doi:10.3390/mi12040424)

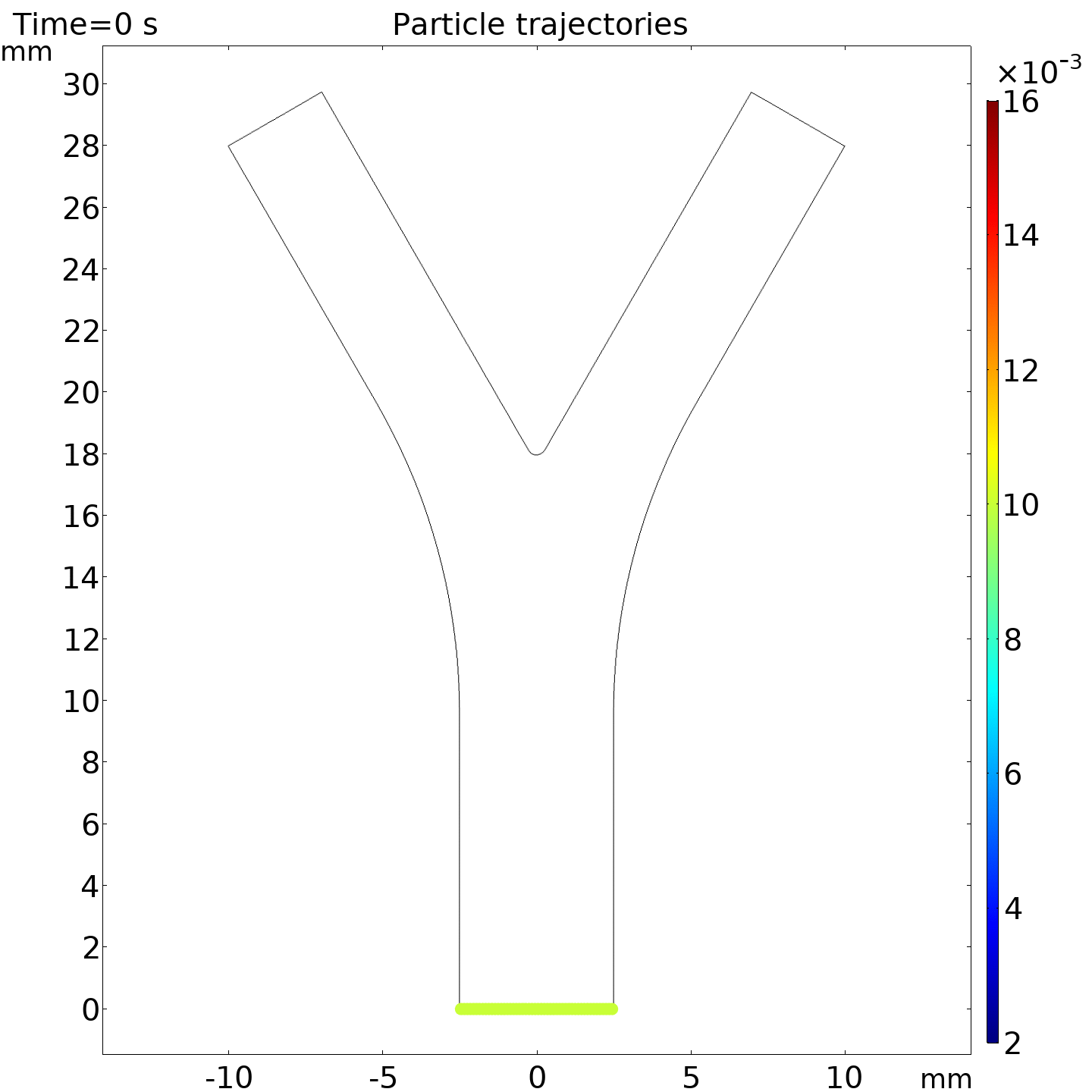

Supplement: Supplementary file 1 [file micromachines-12-00424-s001.zip › Animation Files/Ani_2D_0.gif]

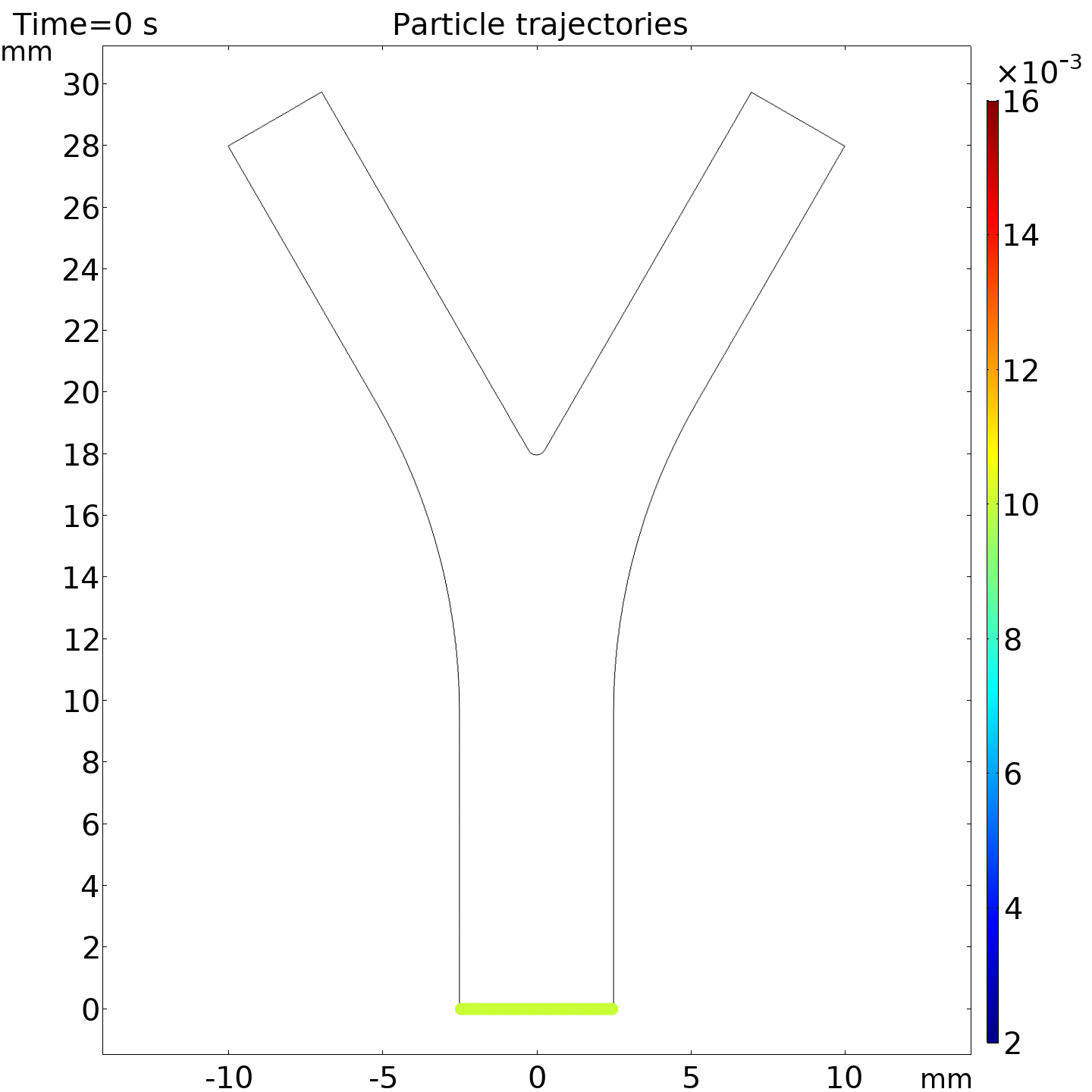

Supplement: Supplementary file 1 [file micromachines-12-00424-s001.zip › Animation Files/Ani_2D_1.gif]

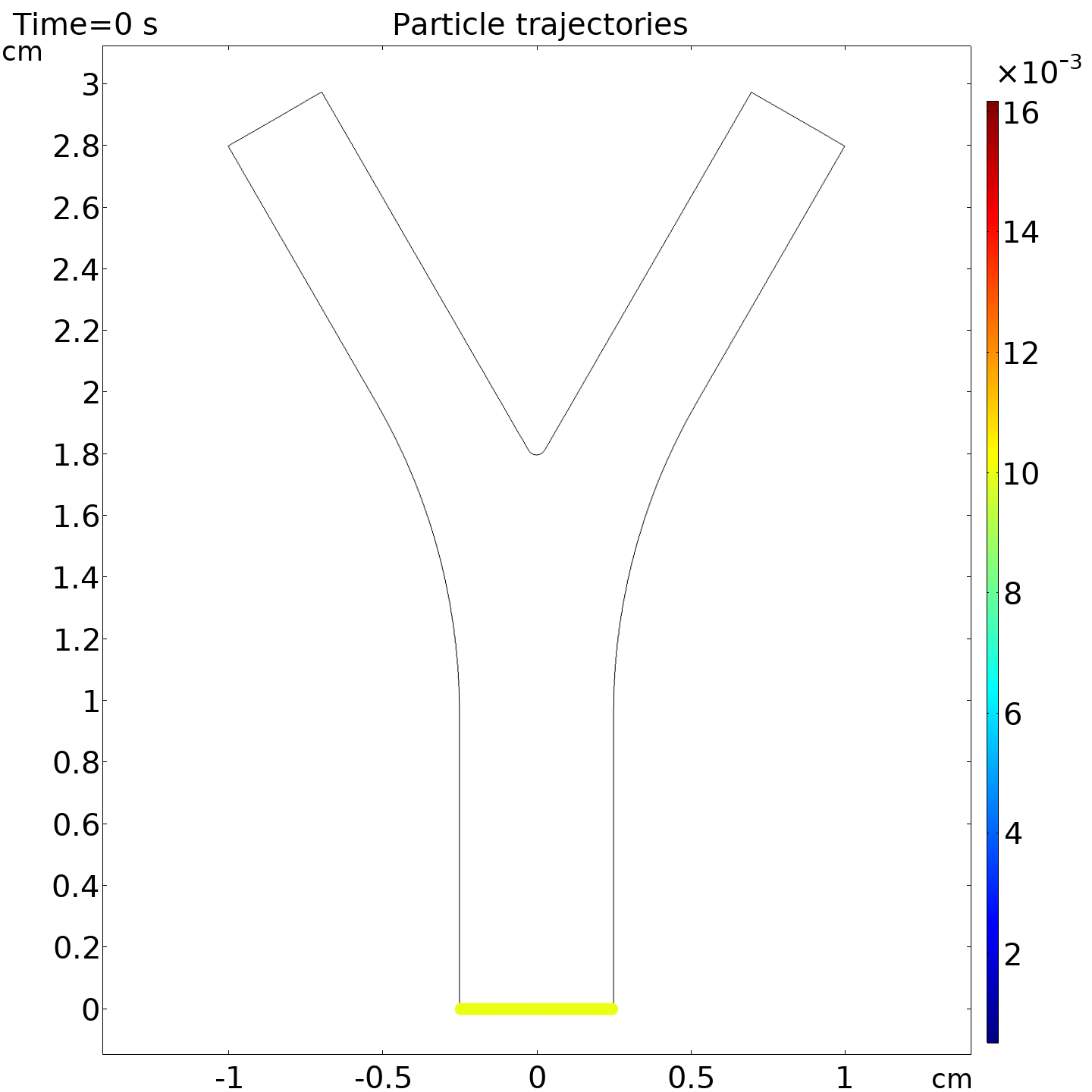

Supplement: Supplementary file 1 [file micromachines-12-00424-s001.zip › Animation Files/Ani_2D_2.gif]

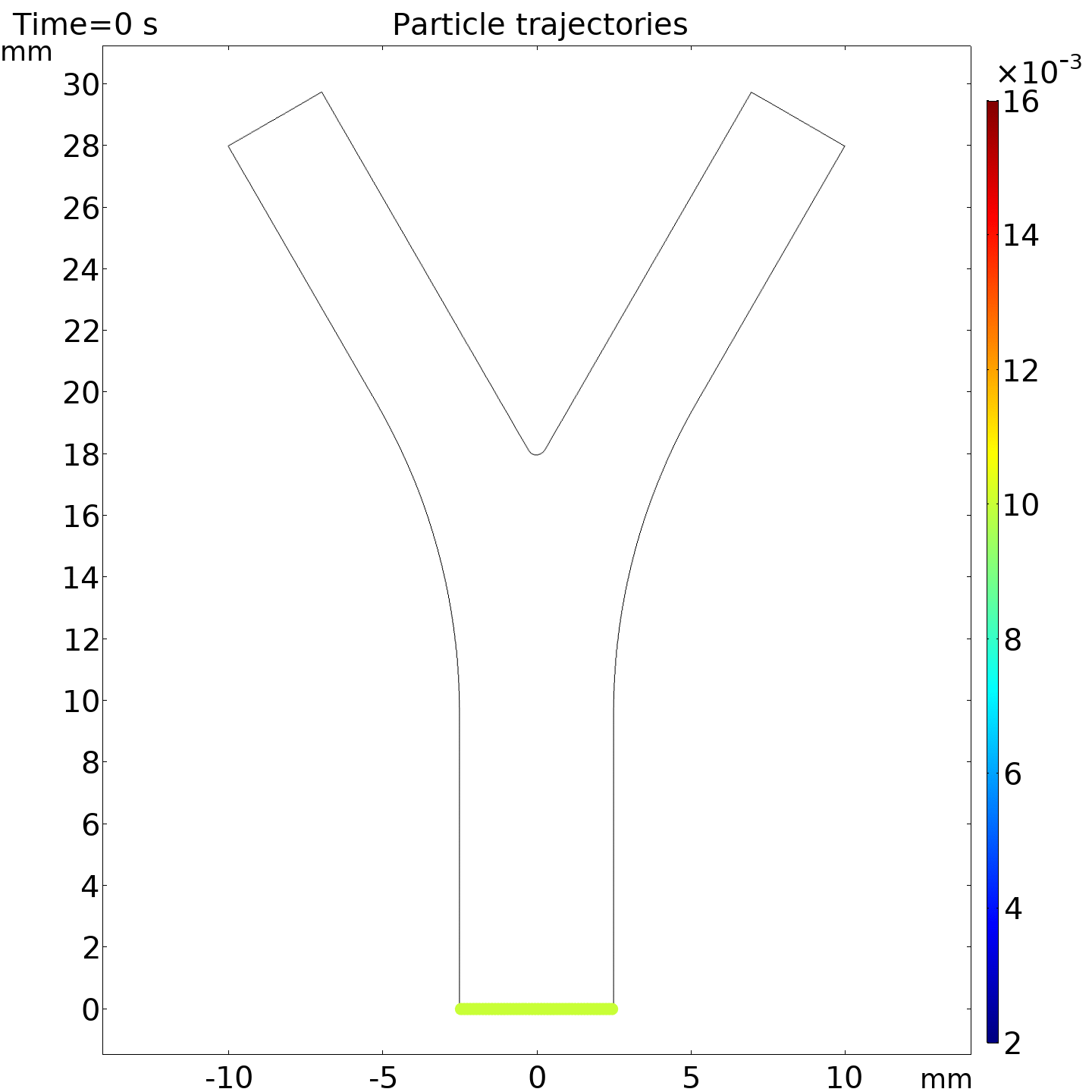

Supplement: Supplementary file 1 [file micromachines-12-00424-s001.zip › Animation Files/Ani_2D_3.gif]

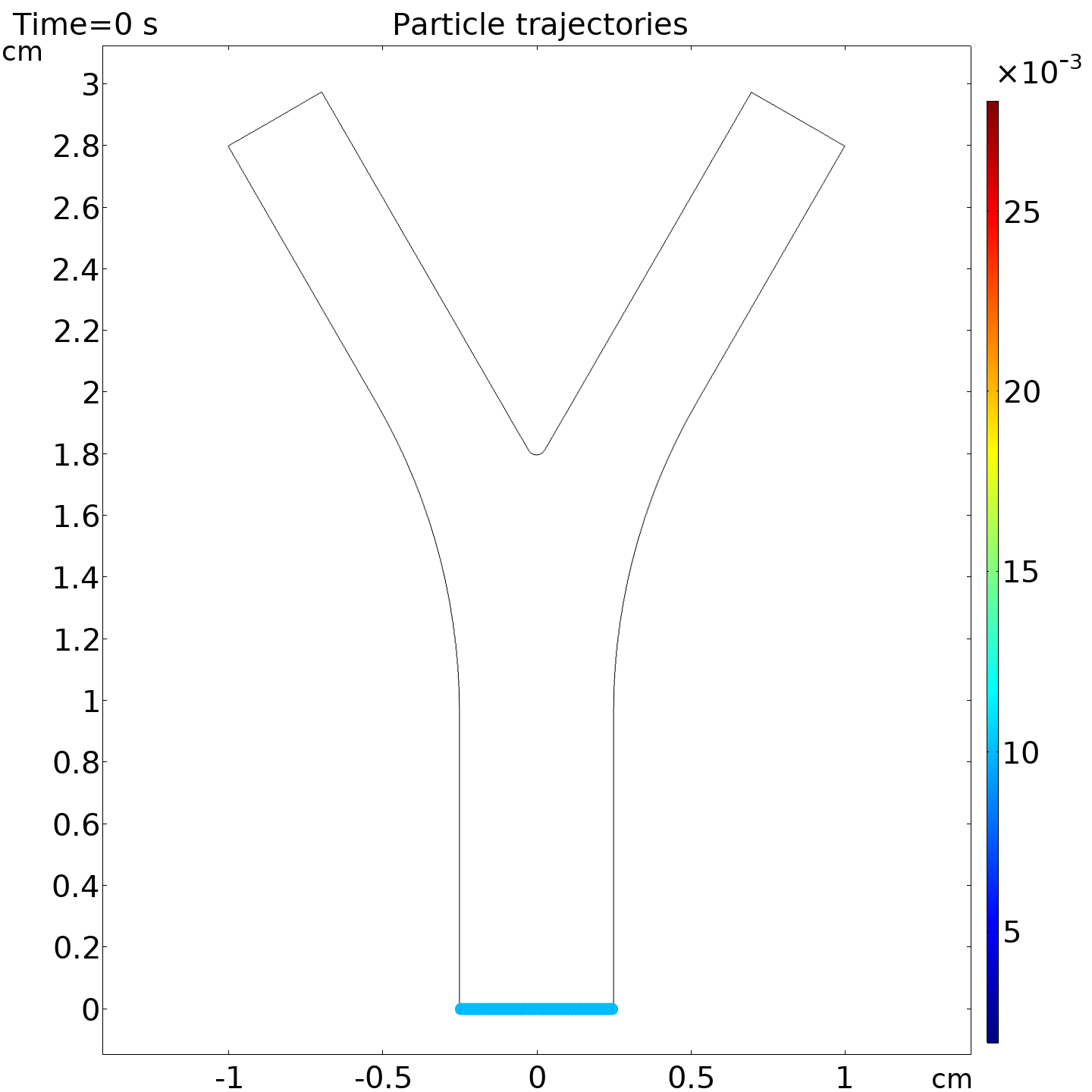

Supplement: Supplementary file 1 [file micromachines-12-00424-s001.zip › Animation Files/Ani_2D_4.gif]

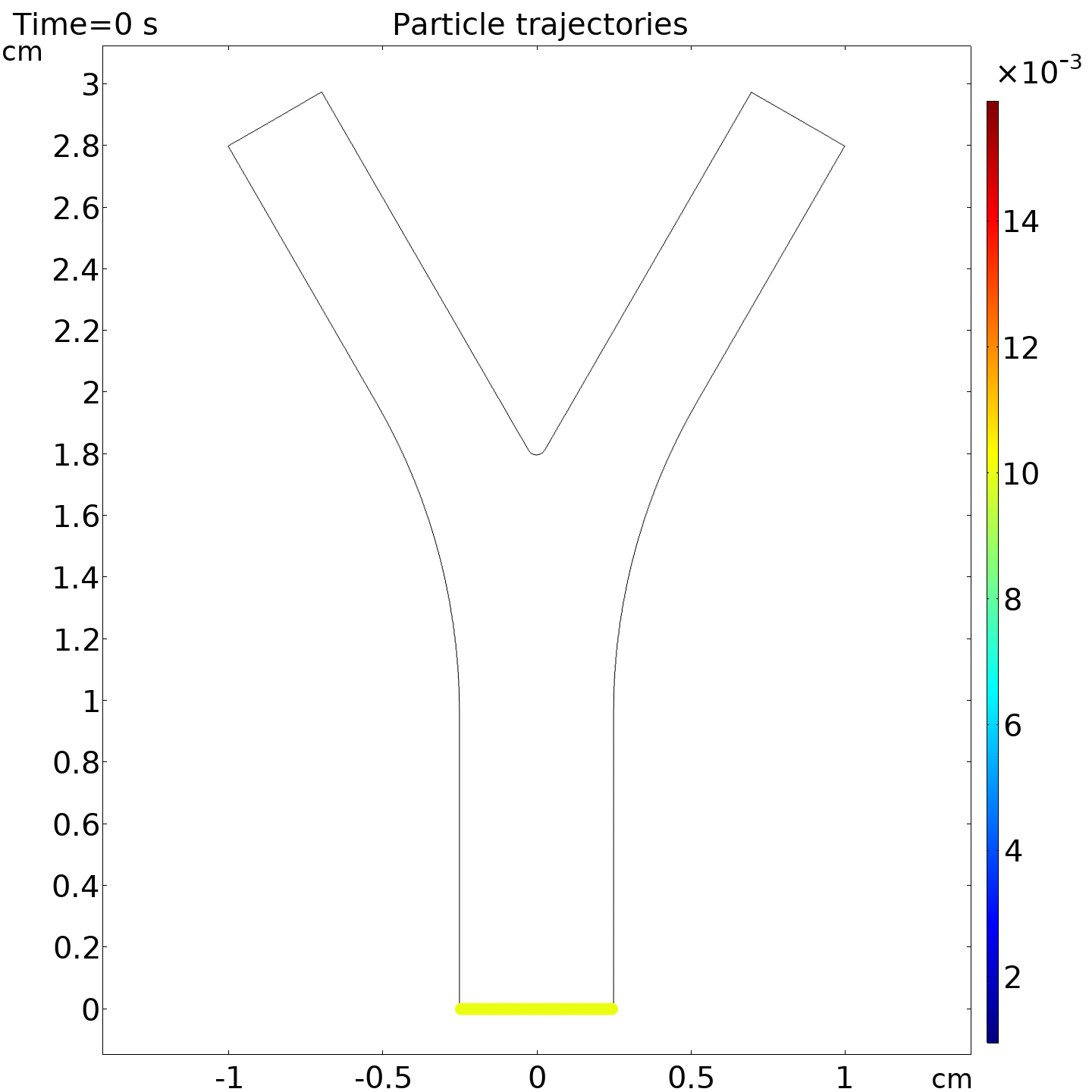

Supplement: Supplementary file 1 [file micromachines-12-00424-s001.zip › Animation Files/Ani_2D_5.gif]

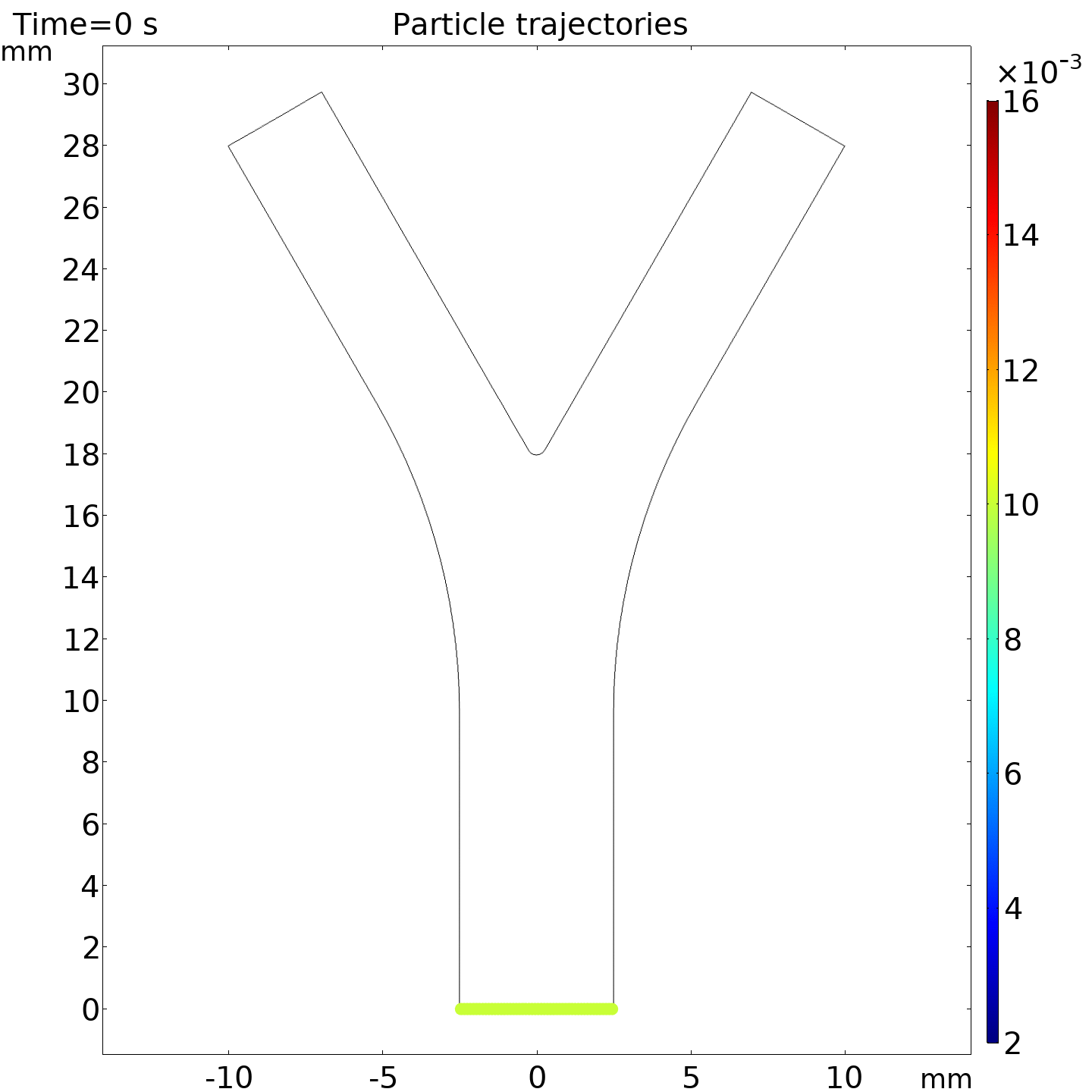

Supplement: Supplementary file 1 [file micromachines-12-00424-s001.zip › Animation Files/Ani_2D_6.gif]

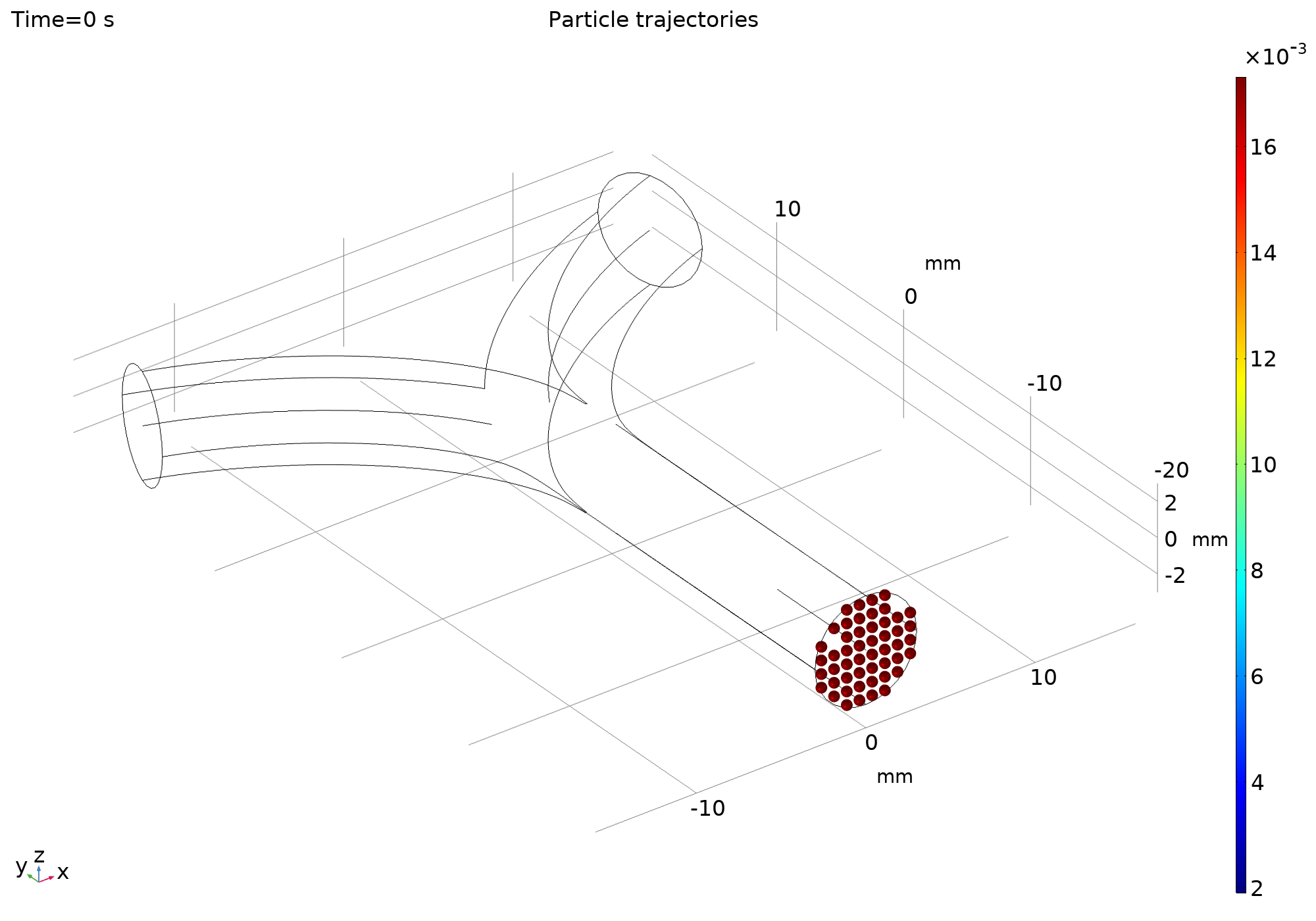

Supplement: Supplementary file 1 [file micromachines-12-00424-s001.zip › Animation Files/Ani_3D_0.gif]

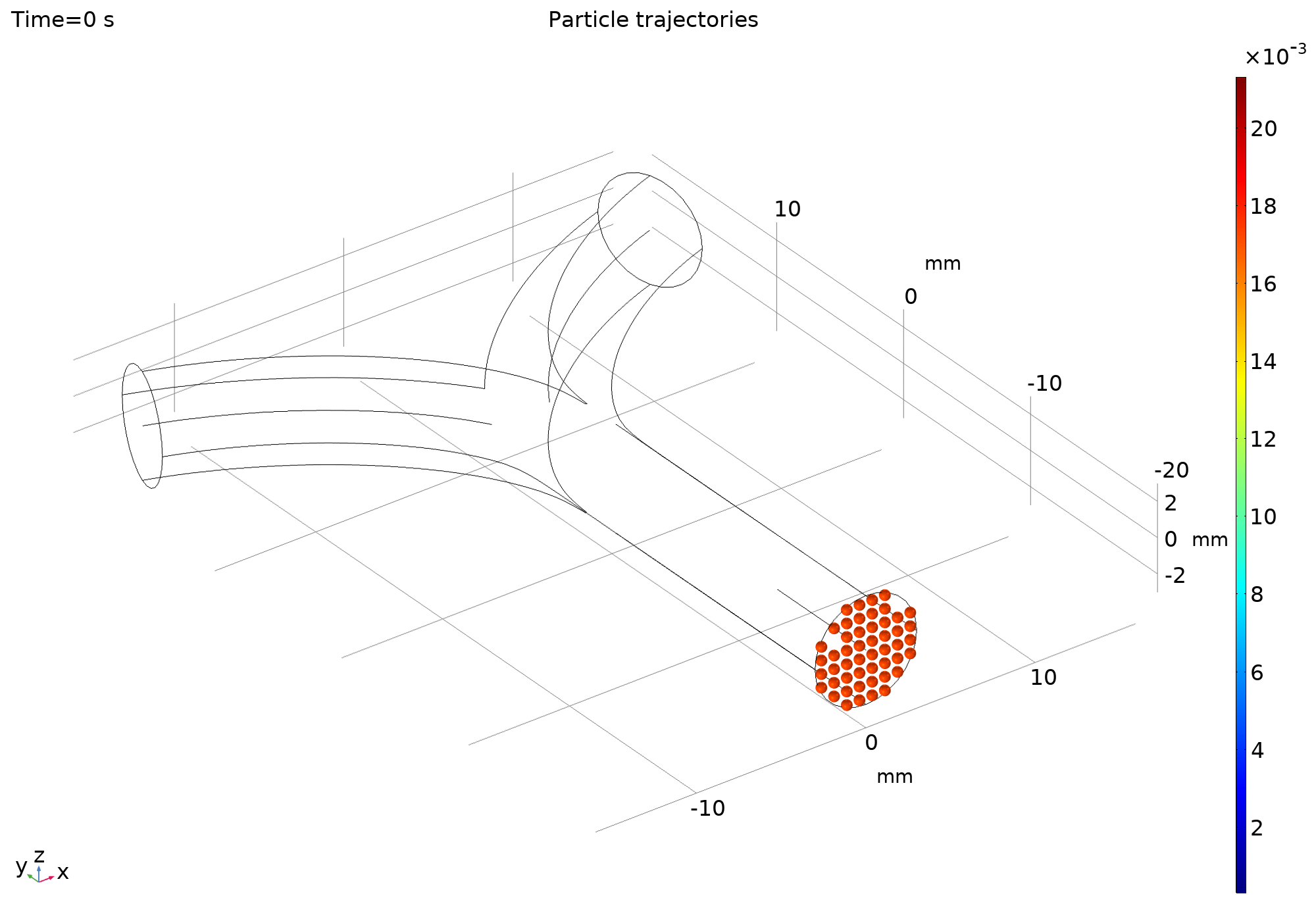

Supplement: Supplementary file 1 [file micromachines-12-00424-s001.zip › Animation Files/Ani_3D_1.gif]

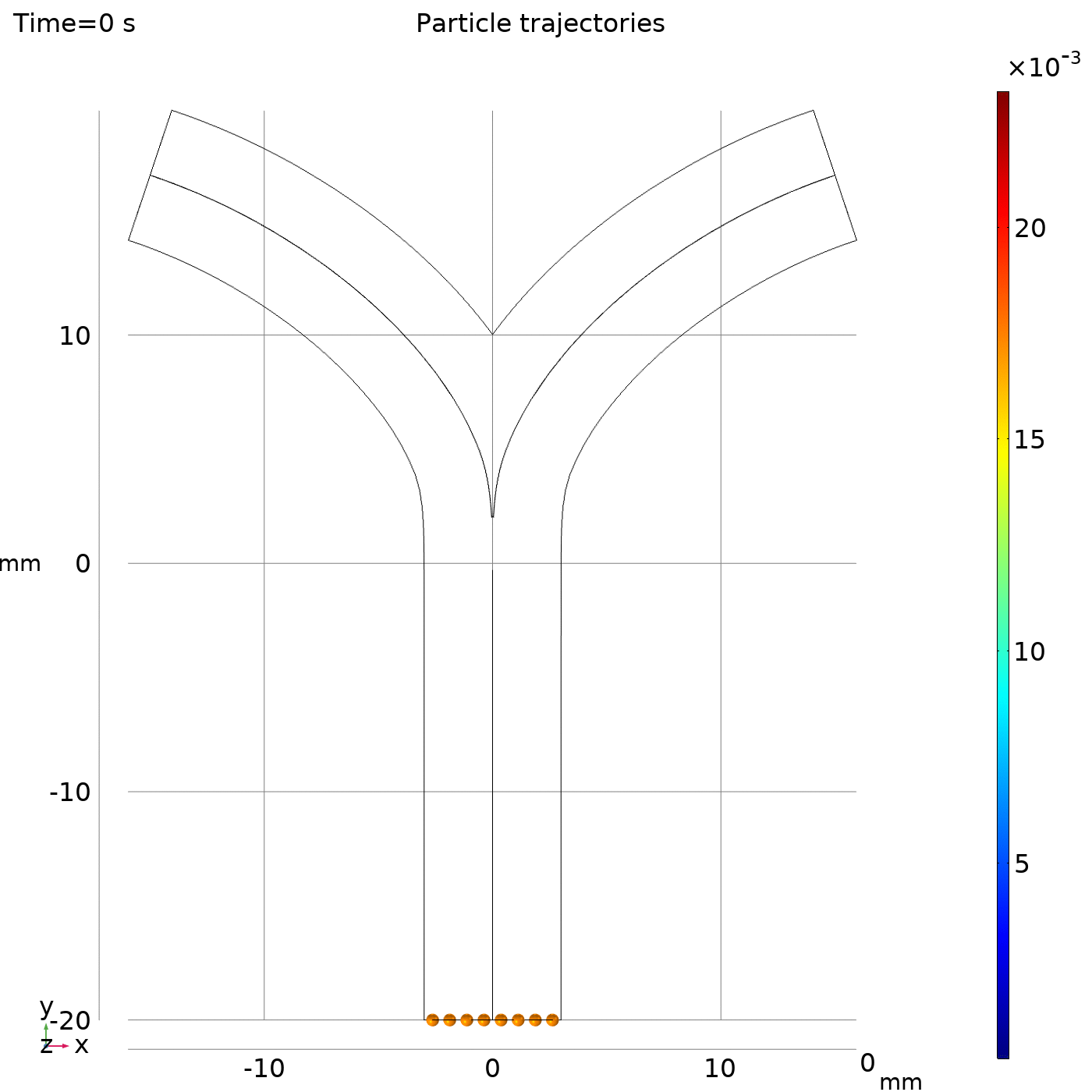

Supplement: Supplementary file 1 [file micromachines-12-00424-s001.zip › Animation Files/Ani_3D_10.gif]

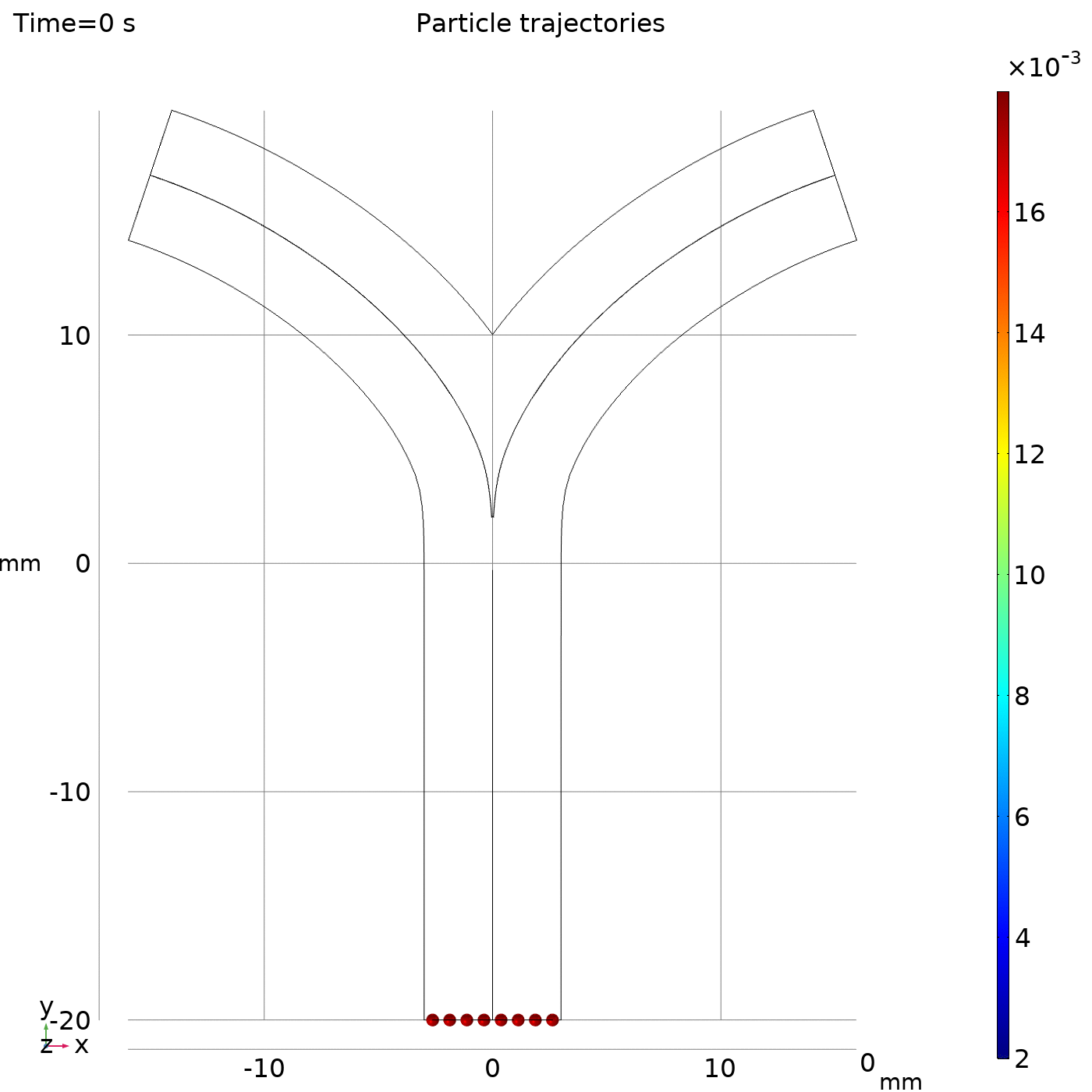

Supplement: Supplementary file 1 [file micromachines-12-00424-s001.zip › Animation Files/Ani_3D_11.gif]

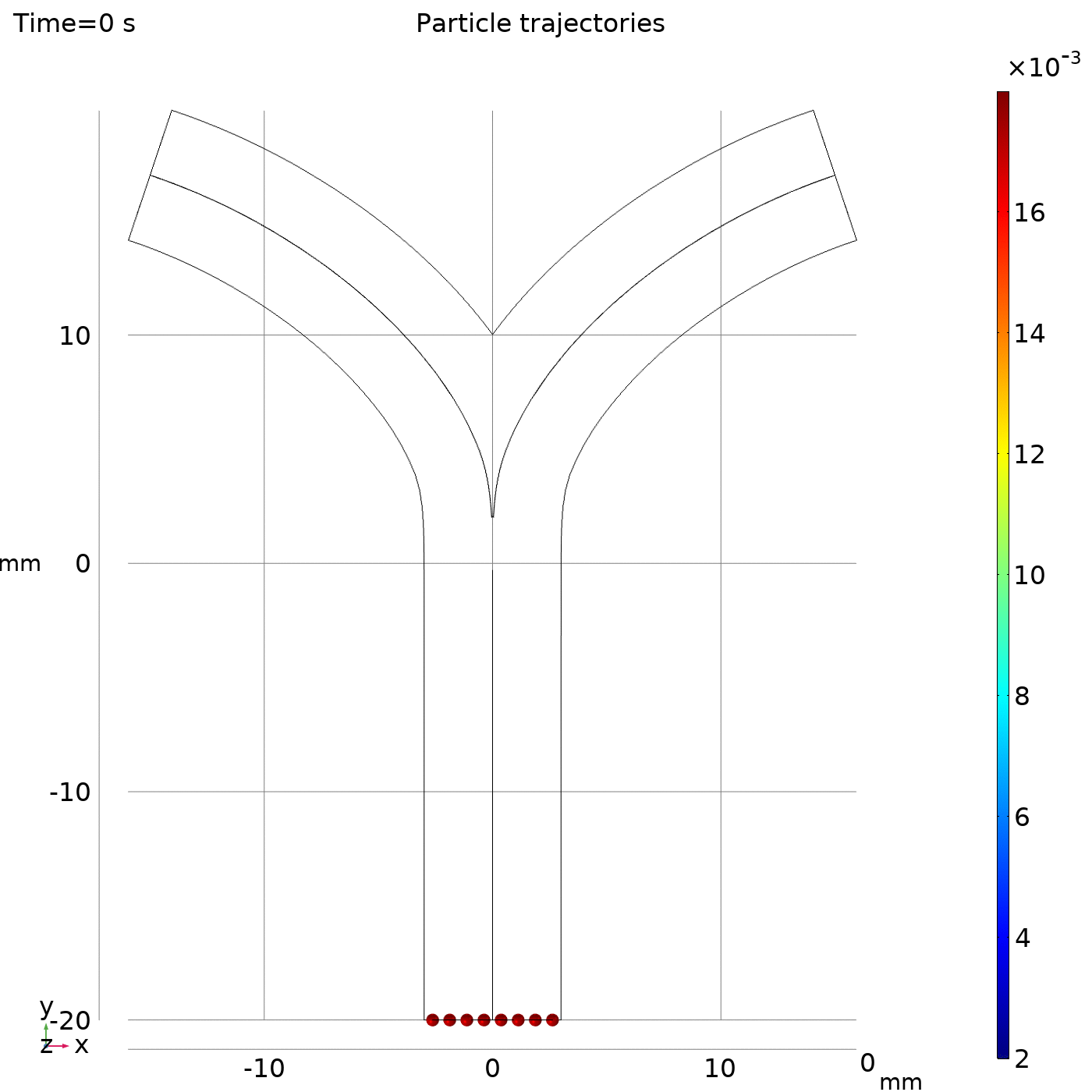

Supplement: Supplementary file 1 [file micromachines-12-00424-s001.zip › Animation Files/Ani_3D_12.gif]

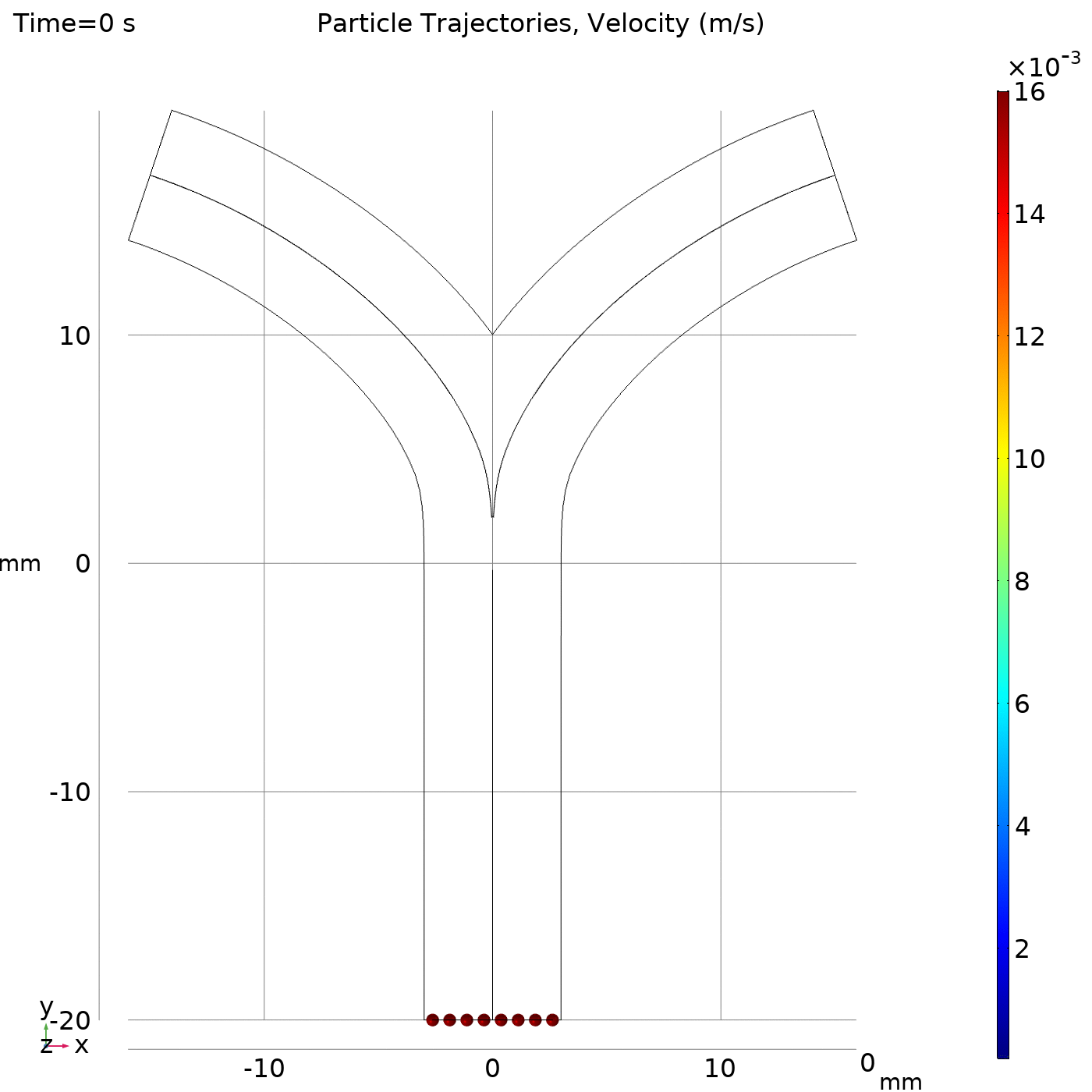

Supplement: Supplementary file 1 [file micromachines-12-00424-s001.zip › Animation Files/Ani_3D_2.gif]

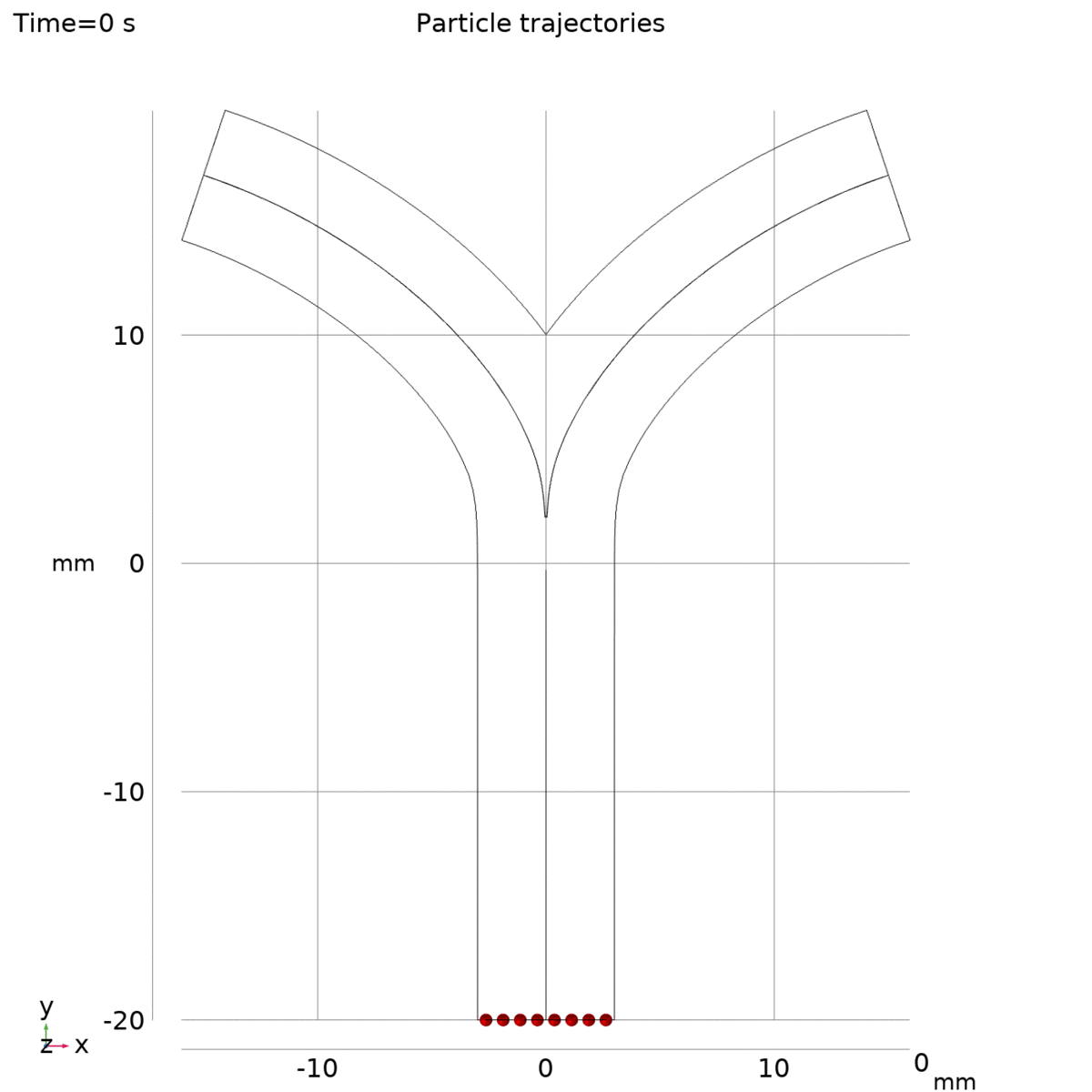

Supplement: Supplementary file 1 [file micromachines-12-00424-s001.zip › Animation Files/Ani_3D_3.gif]

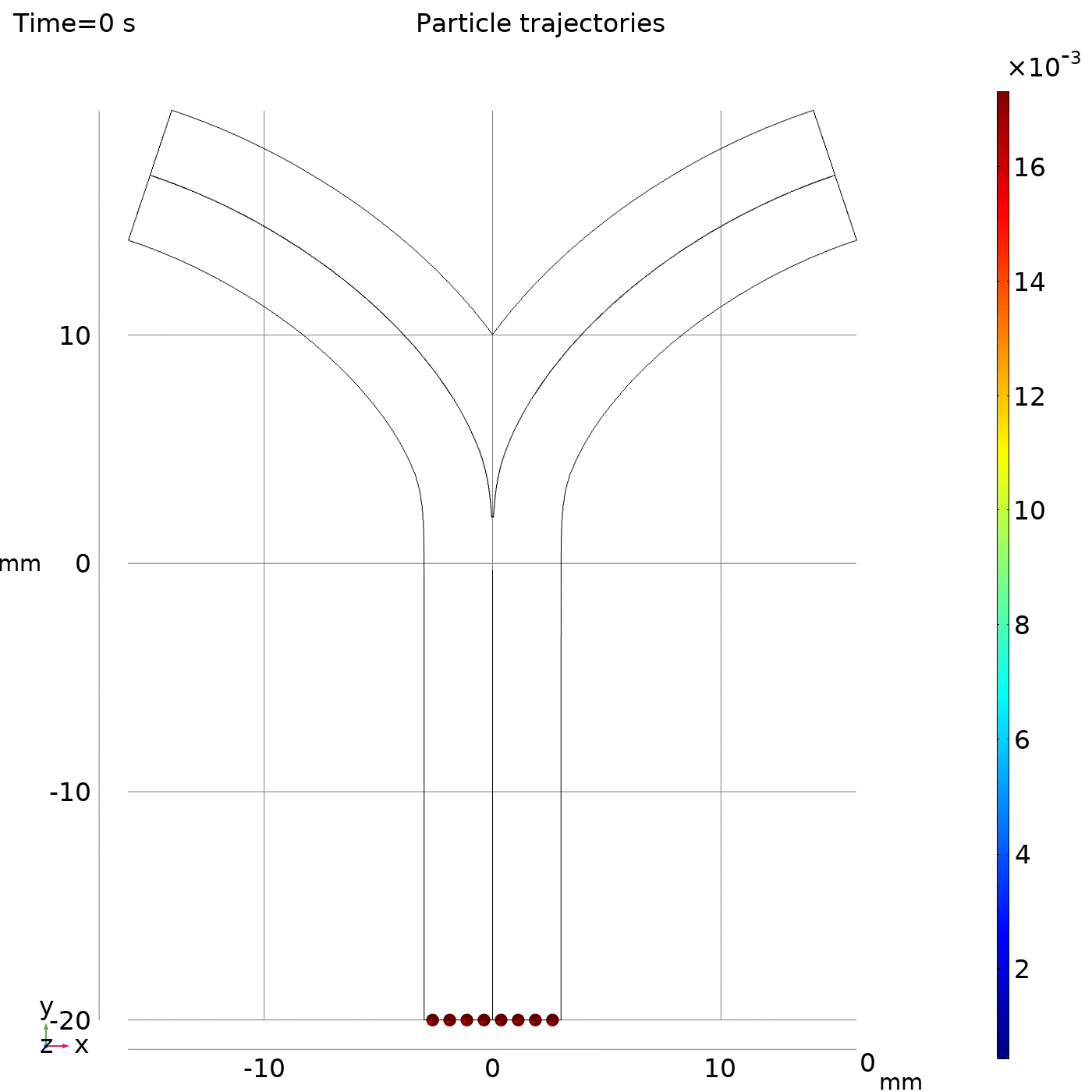

Supplement: Supplementary file 1 [file micromachines-12-00424-s001.zip › Animation Files/Ani_3D_4.gif]

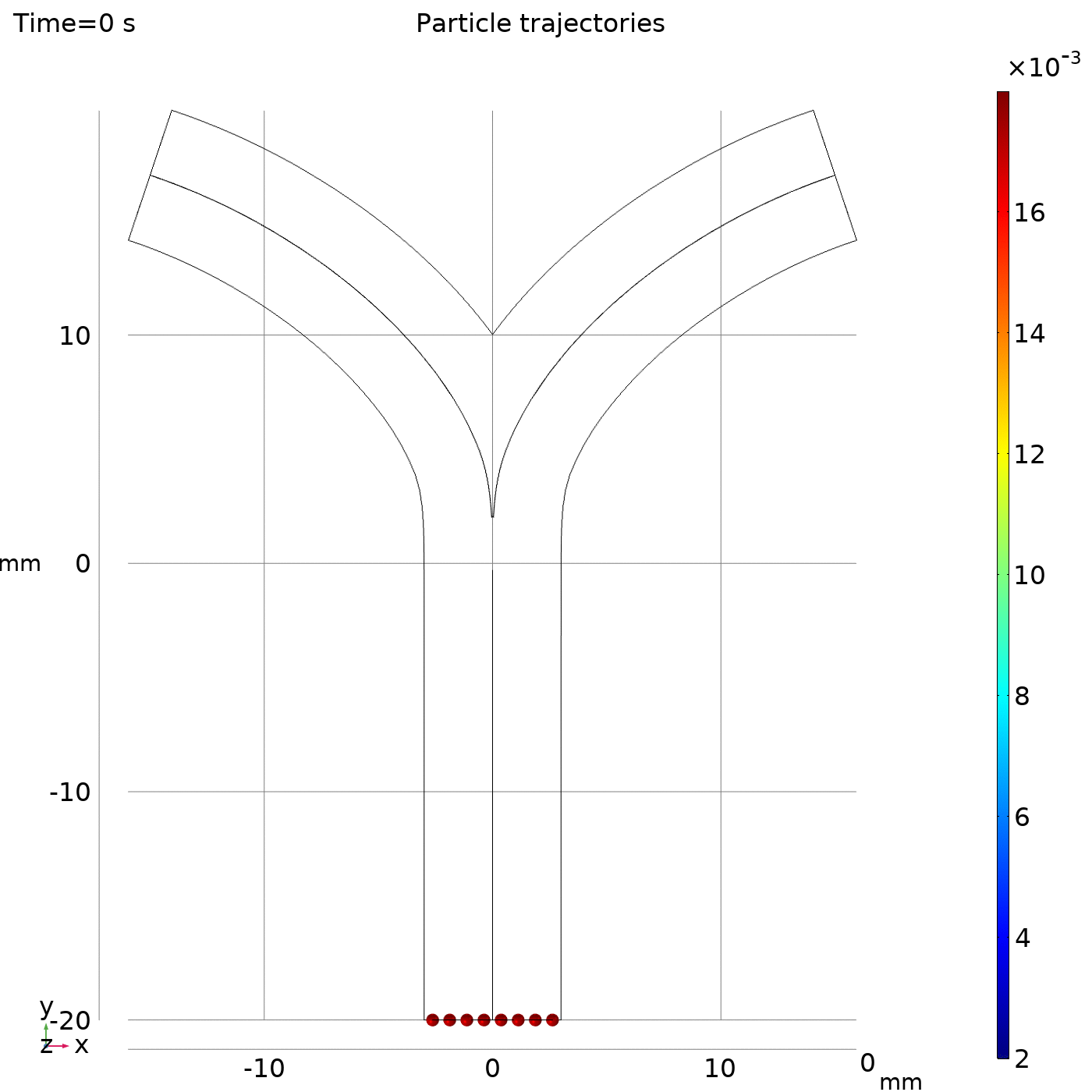

Supplement: Supplementary file 1 [file micromachines-12-00424-s001.zip › Animation Files/Ani_3D_5.gif]

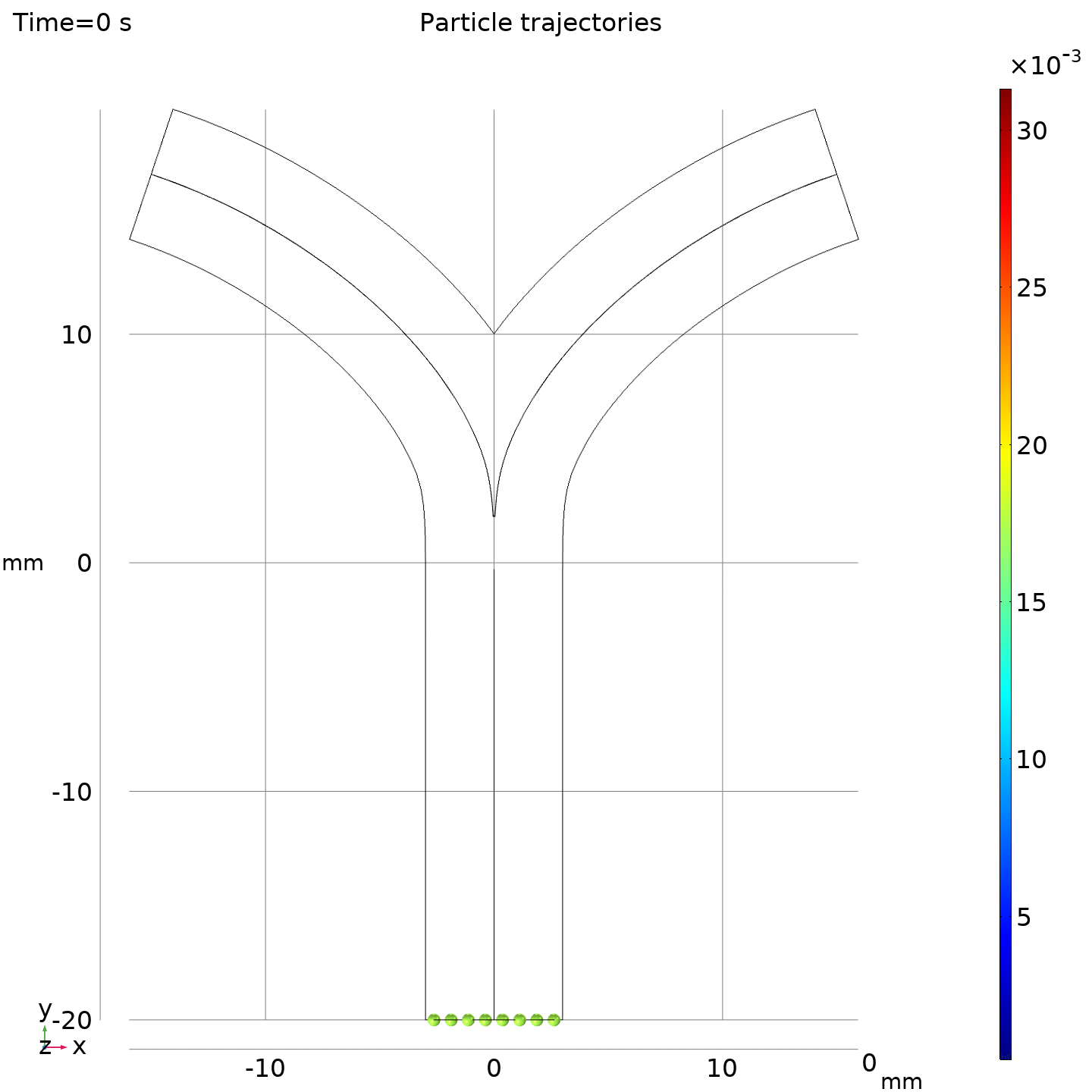

Supplement: Supplementary file 1 [file micromachines-12-00424-s001.zip › Animation Files/Ani_3D_6.gif]

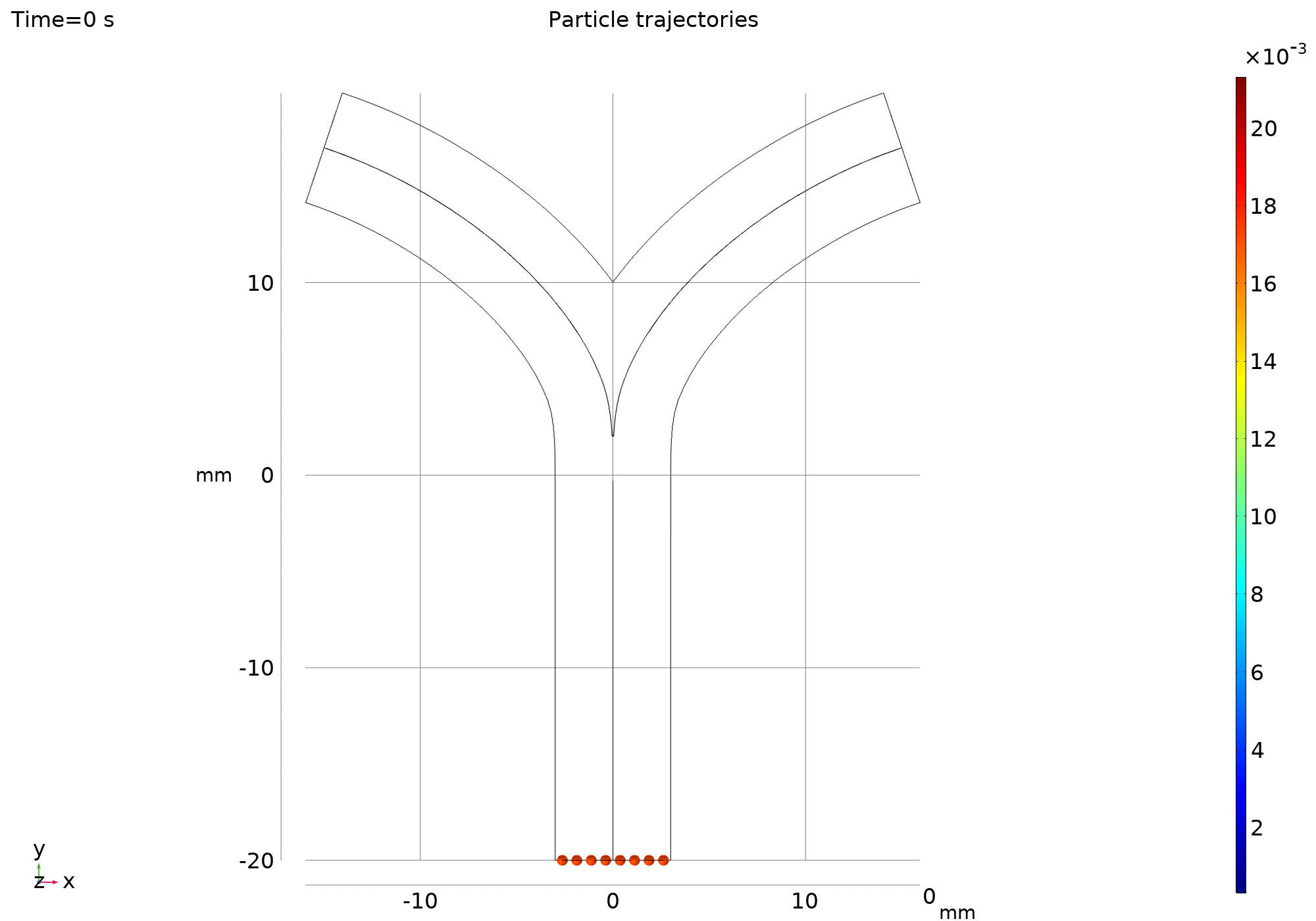

Supplement: Supplementary file 1 [file micromachines-12-00424-s001.zip › Animation Files/Ani_3D_7.gif]

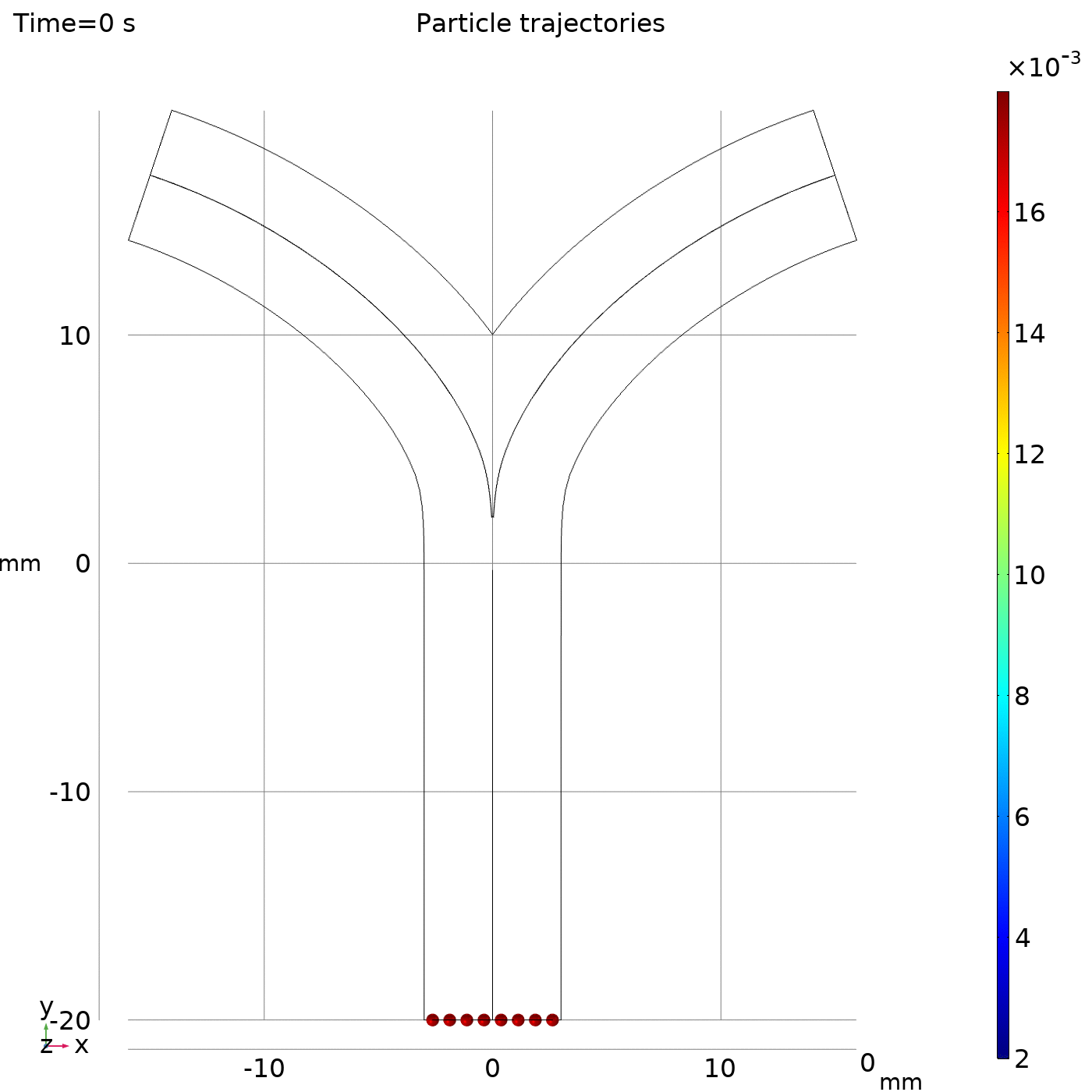

Supplement: Supplementary file 1 [file micromachines-12-00424-s001.zip › Animation Files/Ani_3D_8.gif]

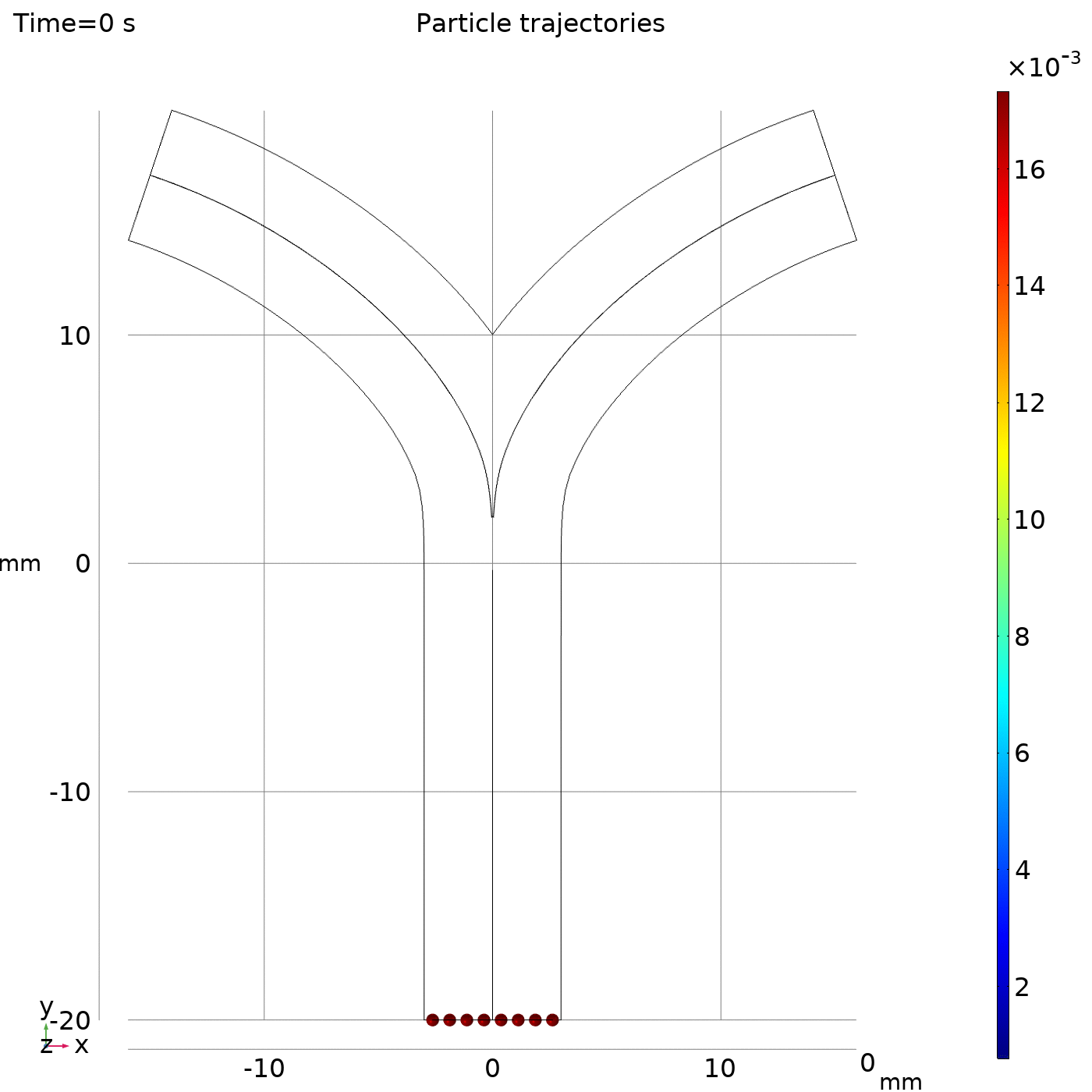

Supplement: Supplementary file 1 [file micromachines-12-00424-s001.zip › Animation Files/Ani_3D_9.gif]
